# Family responses to resource scarcity

**DOI:** 10.1007/s11747-022-00882-7

**Published:** 2022-06-30

**Authors:** A. R. Shaheen Hosany, Rebecca W. Hamilton

**Affiliations:** 1 School of Business and Management, Royal Holloway, University of London, TW20 0EX Surrey, UK; 2 McDonough School of Business, Georgetown University, DC 20057 Washington, USA

**Keywords:** Resource scarcity, Life events, family consumption, Family decision-making, Consumption adjustment, Resource investment

## Abstract

Resource scarcity, manifested through limited time, money or space, is a prevalent aspect of family life. Drawing on depth interviews with 30 families from diverse demographic backgrounds, this study develops a framework to demonstrate how families respond to resource scarcity. Our research examines how multi-dimensional, concurrent and/or consecutive life events, such as job changes, house moves, or childbirth, create a mismatch between available and required resources to trigger situational resource scarcity. We identify different patterns of adjustments in consumption and resource investment over time, based on families’ chronic resources and reliance on support networks. Notably, the greater flexibility afforded by multiple family members is constrained by collective goals, domains of control, tensions and negotiations.

**Supplementary Information:**

The online version contains supplementary material available at 10.1007/s11747-022-00882-7.

## Introduction

Consumers invest resources, such as money, time and space, to achieve their consumption goals. They may allocate part of their pay check to buy new clothes, or spend time cooking a meal. Resource scarcity occurs when consumers lack access to sufficient resources to achieve consumption goals (Hamilton et al., 2019b). Resource scarcity affects consumers at all income levels (Bellezza et al., 2017), and can manifest through limited finances (Paley et al., 2019), time (Kapoor & Tripathi, 2020), or space (Sevilla & Townsend, 2016). To date, studies on resource scarcity focus on individuals (e.g. Mittal et al., 2020), generally portraying a maximising or efficiency perspective (Mullainathan & Shafir, 2013). Individuals respond to resource scarcity by prioritising their most important goals and using their resources more efficiently (Fernbach et al., 2015). Notably, scarcity may prompt individuals to demonstrate immoral (Goldsmith et al., 2018) or selfish behaviours (Roux et al., 2015).

The family is an important unit of production (Becker, 1965) and consumption (e.g. Bettany et al., 2014), with household consumption expenditure representing around 60% of Gross Domestic Product in western economies (OECD, 2021). The family differs from the individual consumer on multiple dimensions. First, family decision making involves reconciling multiple goals (Epp & Price, 2011). A parent’s trip to the supermarket – a superficially individual task – involves fulfilling multiple family members’ needs within budgetary, time and space constraints. During shopping, a parent may consider balancing a partner’s preferences with a child’s in choosing ingredients for a meal, debate the merits of utilising more time to cook a meal from scratch, versus spending more money to buy a partially prepared version, or decide to invest more time to find good deals, substituting time for money (Hoch et al., 1995). Second, shared, family consumption involves interactions (Wu et al., 2021), as families navigate complex, inter-related journeys, ranging from routine meal times (Epp & Price, 2018) to special vacations (Epp & Price, 2011). Finally, multiple members contribute resources to support the family’s consumption of meals (Epp & Price, 2018), vacations (Epp & Price, 2011), child care (Epp & Velagaleti, 2014), pet care (Bettany & Kerrane, 2018), and technology (Bettany & Kerrane, 2016; Nash et al., 2018).

To date, limited research examines how families respond to resource constraints (Hamilton et al., 2019b). From a resource scarcity perspective, Commuri and Gentry (2005) assess how resource allocation within families is prescribed by partners’ relative incomes. Durante et al. (2015) study whether families spend more on sons versus daughters during an economic recession. From a family consumption perspective, studies investigate how occurrences such as childbirth (Thomas & Epp, 2019), divorce (Thompson et al., 2018), single fatherhood (Harrison et al., 2021) or separation (Epp et al., 2014) lead to time, money and space constraints. Despite the resource implications of previous studies, we note the lack of a general theorisation on how families respond to resource scarcity. Accordingly, the primary purpose of this research is to investigate how families respond to money, time and space scarcity, thus extending the resource scarcity literature to families (Hamilton et al., 2019b).

Our work makes several contributions. First, contrary to existing studies that investigate individual responses, we examine how families respond to resource scarcity. Although prior research investigates occurrences that lead to resource scarcity, such as lack of time and other resources for child care (Epp & Velagaleti, 2014) and lack of space and time to handle long distance relationships (Epp et al., 2014), their focus was on these substantive problems rather than on resource scarcity. We identify that the flexibility generated by multiple members prompts consumption and resource trade-offs as families respond to resource constraints. However, the added flexibility is constrained by domains of control (Bettany et al., 2014; Epp & Velagaleti, 2014), negotiations (Cowan et al., 1984) and tensions (Epp & Velagaleti, 2014) as families prioritise collective goals (Epp & Price, 2011).

Second, we examine how a range of family life events lead to situational resource scarcity, which arises in response to specific events. Prior family research recognises childbirth (Bettany et al., 2014; Thomas & Epp, 2019), divorce (Thompson et al., 2018), separation (Epp et al., 2014) or adult children leaving the family home (Hogg et al., 2004) as important events impacting family resources. Building on previous works, our research identifies how a range of concurrent and/or consecutive life events cause transient or prolonged changes in family resources. We categorise life events (e.g. weddings, major illnesses, divorce) leading to situational resource scarcity along multiple dimensions: valence (positive, neutral, negative), duration (transient or prolonged), cause (voluntary or involuntary), and frequency (one-time or recurrent).

Third, our research highlights temporal patterns in family responses to resource scarcity. Hamilton et al. (2019a) suggest that individuals respond to financial scarcity through sequential steps, where they first react and cope - adjust in the short term, and then adapt in the longer term. Analogous to this sequence, we find that in the short term, families respond to resource scarcity primarily by adjusting consumption and becoming more efficient. Families either reduce total consumption or broaden their consideration sets and consume differently (Hill et al., 1998). In the longer term, we observe more substitution between resources. Families substitute time for money through, for example, paid employment and money for space through home improvements. Families also adjust their total resource investment across members. In theorising the temporal nature of family responses to time, money and space scarcity, our study extends Hamilton et al.’s (2019a) framework on individual responses to financial constraints.

Finally, our work examines how families’ chronic resource levels influence their responses to situational resource scarcity. Based on the number of resource providers (adults who provide caregiving and generate income) and the family’s long-term level of resources (based on wealth and income), families face severe, moderate or mild chronic resource scarcity. We observe that across chronic levels of resources, most families experienced time scarcity, primarily related to childcare. However, families facing severe or moderate chronic resource scarcity described more money and space constraints. Families with severe chronic resource scarcity respond to situational scarcity via more consumption adjustment, less resource adjustment, and greater reliance on support networks, including extended family and government services. In contrast, families with mild chronic resource scarcity primarily respond via greater resource investments (e.g. by paying for child care) and less consumption adjustment and demonstrated less reliance on support networks. Families with moderate resources used a mix of responses. To this end, our paper responds to calls for research by Hamilton et al. (2019a) and Goldsmith et al. (2020a) by investigating the interaction between chronic and situational resource scarcity.

## Theoretical background

### Resource scarcity

Prior research distinguishes between situational and chronic resource scarcity. Situational scarcity occurs when resources needed to fulfil specific goals at specific points in time are limited. Chronic scarcity describes resource availability over an extended period of time and may be captured by variables such as income or socioeconomic status (Hamilton et al., 2019b; Goldsmith et al., 2020a). Thus, money can be a scarce resource either when individuals have a small budget to achieve a specific goal (situational scarcity), or when they are permanently living on a low income (chronic scarcity). Similarly, time and space may be limited to fulfil goals at a specific point in time, or they may be limited in the longer term.

Depending on whether studies investigate situational or chronic scarcity, they tend to operationalise scarcity differently (Hamilton et al., 2019a; see Table 1). Situational resource scarcity can be manipulated in lab settings even for consumers who are not experiencing chronic scarcity (e.g. Donnelly et al., 2021; Kapoor & Tripathi, 2020). Proxies for chronic scarcity include childhood socioeconomic status (e.g. Thompson et al., 2020; Wang et al., 2020) or income (e.g. Commuri & Gentry 2005). Although some similarities exist in the effects of situational and chronic scarcity (e.g. focusing on the scarce resource - Mullainathan & Shafir 2013), chronic resource scarcity has a longer-term influence on decision-making.

**Table 1:** Key literature on resource scarcity

Studies	Context	Level of Investigation	Type of Scarcity Studied						Key Findings
			Time	Money		Space	Other^*^	Chronic vs. Situational	
Roux et al. (2015)	Selfish behaviours	Individual	X	X		X	✓	S	Consumers engage in behaviours that advance their own welfare through competitive orientation.
Mehta and Zhu (2016)	Product use creativity	Individual	✓	X		X	✓	S	Scarcity promotes product use creativity as a maximising behaviour.
Xu and Albarracin (2016)	Space constraint & vice products	Individual	X	X		✓	X	S	Smaller space reduces impulsive consumption of vice products and leads to lower consumption of high calorie products.
Goldsmith et al. (2018)	Immoral behaviour	Individual	X	X		X	✓	S	Consumers who adopt a maximising mindset are more likely to engage in immoral behaviours.
Zhu et al. (2019)	Deadlines and goal pursuit	Individual	✓	X		X	X	S	Long deadlines produce adverse effects on goal pursuit through increased procrastination and higher possibility of quitting.
Goldsmith et al. (2020b)	Sustainable product adoption	Individual	✓	X		X	✓	S	Consumers demonstrate a higher interest in sustainable products when their prosocial rather than personal benefits are highlighted.
Hill (2020)	Impoverished consumers	Individual	X	✓		✓	✓	C	Identifies how deprived consumers respond differently to scarcity along stages of the consumer decision journey.
Kapoor and Tripathi (2020)	Consumption of high calories	Individual	✓	X		X	X	S	Time keeping direction influences high calorie food consumption.
Mittal et al. (2020)	Childhood SES & consumer self-confidence	Individual	X	✓		X	X	S	Money scarcity during childhood decreases self-confidence.
Thompson et al. (2020)	Childhood SES and substitution	Individual	X	✓		X	X	C	Consumers from low childhood SES are more likely to engage in substitution.
Wang et al. (2020)	Feasibility & desirability of product choices	Individual	✓	X		X	✓	C	Low childhood SES consumers seek more feasibility than desirability during choice when faced with resource scarcity.
Das et al. (2021)	Pandemics	n/a	✓	✓		✓	✓	S	Pandemics create (i) financial scarcity due to business closures and financial losses (ii) scarcity of essentials - supply chain disruption (iii) relational scarcity - loss of interaction between friends and family
Paley et al. (2019)	Word of Mouth	Interpersonal	✓	X		X	X	S	Financial constraints reduce purchase related word of mouth, but does not reduce propensity to share information online.
Lee-Yoon et al. (2020)	Gift giving	Interpersonal	✓	✓		X	X	S	Gift recipients intending to save money experience more negative emotions than those expecting to save time.
Donnelly et al. (2021)	Rejecting social invitations	Interpersonal	✓	✓		X	X	S	Excuses of time, rather than money constraints reduce trust, interpersonal closeness and helping behaviours.
Commuri and Gentry (2005)	Resource allocation in families with wives earning higher income	Family	X	✓		X	X	C	Joint pools of money used for routine expenses, and separate pools for other expenses.
Durante et al. (2015)	Spending on daughters/sons	Family	X	✓		X	X	S	Poor economic conditions (financial scarcity) favours spending on daughters versus sons.
This Study	Family responses	Family	✓	✓		✓	X	S & C	Families as social groups, respond to multiple concurrent and/or consecutive scarcity-induced life events, by adjusting consumption or resource investment. Responses occur within an overall framework of family interactions and characteristics and support networks.

^*^ Includes reminders of resource scarcity, commodity scarcity (e.g. gasoline, sugar, water, wheat, electricity); **✓** indicates resource implied in study; C denotes chronic scarcity and S situational scarcity

Situational and chronic resource scarcity may have distinct effects on consumer journeys (Hamilton et al., 2019b). For example, studies have demonstrated that short term time scarcity due to deadlines influences goal pursuit (Zhu et al., 2019) and that space constraints in hypothetical shopping tasks influence consumption of vice products (Xu & Albarracin, 2016). Consumers facing chronic resource scarcity exhibit long-lasting differences in choice behaviour (Griskevicius et al., 2011). Chronic resource scarcity may lead to lower self-confidence (Mittal et al., 2020) and consumer decisions based on feasibility rather than desirability (Wang et al., 2020). Although chronic resource scarcity may reduce willingness to delay gratification when consumers make choices (Griskevicius et al., 2013), there is evidence that consumers with low childhood socioeconomic status are willing to wait longer for a chosen alternative and show less negative emotional reactions as they wait (Thompson et al., 2020).

Table 1 illustrates that to date, studies on situational and chronic resource scarcity focus mostly on individual responses. Exceptions include Paley et al. (2019), who assess the effects of financial constraints on sharing word of mouth with other consumers, Lee-Yoon et al. (2020) who study the effects of scarcity on gift giving, and Donnelly et al. (2021) who research how revelations of time, rather monetary scarcity, reduce trust. From a family perspective, Commuri and Gentry (2005) and Durante et al. (2015) investigate the impact of financial scarcity on family decision making and consumption. We next turn to the scholarship on family consumption, and relationship between family consumption and resource scarcity.

### Family consumption

Consistent with Epp and Price (2011), we define families as “networks of people who share their lives over long periods of time bound by ties of marriage, blood, or commitment, legal or otherwise, who consider themselves as family and who share a significant history and anticipated future of functioning in a family relationship” (Galvin et al., 2004, p. 6). Family consumption refers to purchases accomplished by any member(s), for use by the family (Delphy & Leonard, 1980). Table 2 summarizes key research on family consumption, highlighting the contexts, resources studied and limitations with respect to a generalized understanding of family responses to resource scarcity.

**Table 2:** Comparison of key family consumption research** (**with implications for resource scarcity)

Studies	Context or Life Event	Research Focus	Participants	Resource Implications			Key Findings	Main Limitations/Gaps (in relation to resource scarcity)
				Time	Money	Space		
Hogg et al. (2004)	Empty nest household	Family transitions	Mothers of empty nesters	✓	✓	✓	Energy, emotions, juggling, routines, control, companionships, rituals are involved in parenting.	Focuses on only one life transition (children moving out); does not investigate constraints through the lens of resource scarcity.
Epp and Price (2008)	Family identity in consumption	Individual, relational and collective identity	n/a (conceptual)	✓	✓	X	Communication forms, and marketplace resources combine to manage conflicting identities.	Omits the exploration of multiple family identities via resource scarcity theory.
Epp and Price (2011)	Family vacations	Customer network (family) identity goals	Parents & children	X	X	X	Typology of identity goals to establish how families accomplish synergies with market offerings.	Excludes a discussion of resource constraints in maintaining synergy between multiple family goals.
Bettany et al. (2014)	Technology & fatherhood	Role of technology during transition to fatherhood	Fathers of new-borns	X	X	X	Consumption of technology becomes increasingly important across the transition from pre, through to early fatherhood.	Focuses on only one life transition (new fatherhood); overlooks time, money and space implications of technologies.
Epp et al. (2014)	Long distance family practices	Role of brands and technology	Parents & Children	✓	X	✓	Generates understanding on the use of technology to bring together families separated via geographical distances.	Highlights tensions of time and space constraints only; focuses on family separations as one form of life transition.
Epp and Velagaleti (2014)	Child care services	Outsourcing	Parents of children under 18	✓	✓	X	Combination of parent versus outsourced child care depends on level of control, intimacy and substitutability parents wish.	Stresses time and money constraints only, on one aspect of family decision-making (child care).
Karanika and Hogg (2016)	Downwardly mobile consumers, intergenerational support and sharing	Ambivalence in maintaining family ties	Family Members	X	✓	✓	Ambivalence and inter-generational support often lead to conflict based on family identity.	Centres on some aspects of resource scarcity, such as finances and space; disregards time scarcity and does not utilise the lens of resource scarcity.
Banister et al. (2016)	Young mothers on low incomes	Response to parenting challenges	Young mothers	X	✓	X	Reframe mothers’ priorities based on financial constraints	Focusses on financial constraints only
Bettany and Kerrane (2016)	Child surveillance technology	Parent – child relationships	Parents	X	X	X	Examines parent-child relationships, child welfare and privacy.	Ignores resource restraints in acquiring and using surveillance technology.
Epp and Price (2018)	Macro-environmental disruptions	Feeding the family	n/a (conceptual)	✓	✓	X	Emphasizes changes in feeding the family such as participation of dads and innovation in food systems	Tacit implications of time and money constraints only.
Bettany and Kerrane (2018)	Pet stock keeping as hobby	Negotiations, resistances & agencies	Parents of children under 18	X	X	X	Illustrates a range of parental behaviours, motivations, activities and children responses towards consumption of pet stock.	Disregards resource implications of keeping pet stock.
Davis et al. (2018)	Food & health	Identity and gendered caring	n/a (conceptual)	✓	✓	X	Reveals the social class, emotional and gendered work involved in feeding the family.	Alludes to constrained time and money only.
Nash et al. (2018)	Console gaming	Family togetherness, consumption	Parents of children under 18	✓	X	X	Family togetherness through gaming is unsustainable; relational bonding is more realistic.	Neglects money and space considerations involved in gaming.
Thompson et al. (2018)	Divorce	Pre-divorce lifestyle impact on consumption	Mothers of children under 17	✓	✓	✓	Lifestyle discontinuities lead to consumption that insulate structurally imposed socio-economic constraints.	Concentrates only on divorce as a life event; implicit reference made to time, money and space constraints.
Thomas and Epp (2019)	Child birth	Planning & habituation of new practices	Parents of new-borns	✓	✓	X	Documents the processes through which new parents realign their planned baby rearing practices based on obstacles encountered.	Focuses on only one life transition (child birth); finances allow access to parenting resources; support network implied.
Harrison et al. (2021)	Single father households	Children socialisation in household tasks	Dads and children (all age groups)	✓	X	X	Household resource gaps and men gender identity lead to six children socialisation processes: entrustment, entrainment, education, emprise, estrangement, elevation.	Mostly suggests time scarcity for household labour due to presence of only one parent; looks into the resource constraint of one family type.
Kerrane et al. (2021)	Brexit	Mothers’ prepping behaviour	Mothers	✓	✓	✓	Discusses how mothers achieve and maintain their survivalist identity by hiding the prepping behaviours to maintain family safety.	Addresses only one scarcity-inducing event; infers time, money and space scarcity due to stocking.
This Study	Previous (any) event recalls	Family responses to resource scarcity	Parents of dependent children	✓	✓	✓	Investigates how families as a collective respond to resource scarcity.	Assesses a range of life events leading to resource scarcity; investigates family decision making through the lens of resource scarcity; analyses time, money and space resource implications.

✓ indicates resource implied in study

Studies of family consumption concentrate primarily on western, developed markets (Thomas & Epp, 2019; Nash et al., 2018), and traditional nuclear structures. Research often focuses on critical periods during the life course, such as childbirth (Bettany et al., 2014; Thomas & Epp, 2019), early childhood (Epp & Velagaleti, 2014), children leaving the family home (Hogg et al., 2004), or divorce (Thompson et al., 2018). Such occurrences or disruptions command changes to routines, often requiring changes in family schedules and plans.

When families engage in shared consumption, from spending money on vacations to devoting time watching TV shows or talking, all or some family members negotiate to fulfil individual, relational (e.g. sibling to sibling) or collective goals (Epp & Price, 2011) using the family’s time, money and space. Because families are made up of multiple individuals, there may be conflict and negotiation among individual, relational and family goals (Epp & Price, 2011). For example, a family may have entertainment as a goal for a vacation. While a beach resort holiday may be ideal for parents and toddlers, teenagers may consider a mountain hike, or city break, more thrilling. Conflicting goals across individuals or relational units lead to family negotiations (Spiro, 1983; Qualls, 1988). Families either prioritise some goals, seek to achieve multiple goals through parallel activities, or partition into groups with separate goals (Epp & Price, 2011).

When family members have conflicting goals, their relative resources and roles determine how conflict is resolved. An early model of family decision making, Household Production Theory (Becker, 1965), conceptualises the family as a production unit combining market-purchased goods with labour to maximize the family’s collective benefits in cleaning, feeding, educating, and entertaining its members. In contrast, bargaining models explicitly incorporate influence and negotiation. The Resource Theory of Family Power (Blood & Wolfe, 1960) contends that the most influential members of a family are those with more resources. Depending on whether a husband or wife controls more resources, he or she is more influential (Commuri & Gentry, 2005). Parents, as figures of authority (e.g. Childers & Rao 1992), handle most resource generation, giving them more influence over resource allocation.

Relative Investment Theory (Davis, 1976) suggests that individual motivation in specific domains will lead to higher influence. Relevant power bases (Blood & Wolfe, 1960; French & Raven, 1959), such as expertise, also shape domains of control. A parent who is expert at cooking will exercise more control on food shopping, while another, over home maintenance. Single parent, same sex, and blended families may use different decision-making processes. Families with support from the extended family (Lien et al., 2018; Karanika & Hogg, 2016) have more flexibility and resources to pool together and share duties, despite loyalties and generational conflicts (Engstrom, 2012; Waites, 2009).

### Families and resource scarcity

When individuals encounter resource scarcity, they reduce consumption or substitute between resources (Hamilton et al., 2019b). Consumers substitute cheaper products (e.g. private labels) for expensive ones (e.g. national brands), or use products more creatively (Mehta & Zhu, 2016). Scarcity may also prompt individuals to demonstrate immoral (Goldsmith et al., 2018) or selfish behaviours (Roux et al., 2015).

In families, time scarcity in dual income earning households leads to child care outsourcing (Epp & Velagaleti, 2014), improvisation in meal planning, food shopping, cooking tasks and shifting gender roles (Epp & Price, 2018). Families bridge space due to long distances through the use of technology (Epp et al., 2014). Single dads socialise their children into household tasks, primarily due to time constraints imposed by having only one parent (Harrison et al., 2021).

As illustrated by these examples and Table 2, previous research tends to focus on either a single form of resource scarcity (financial scarcity, see Commuri & Gentry 2005; Durante et al., 2015; time scarcity, see Harrison et al., 2021; Epp & Velagaleti, 2014) or a single event that generates resource scarcity (childbirth – Thomas & Epp, 2019; new fatherhood – Bettany et al., 2014; divorce or widowhood, see Harrison et al., 2021, Thompson et al., 2018; Brexit - Kerrane et al. 2021; pandemic, see Das et al., 2021). There is a general lack of theorisation on how families with dependent children respond to resource scarcity. To better understand how families respond to time, space and financial scarcity, we conducted a series of semi-structured depth interviews with parents. More specifically, we investigate: (a) What triggers resource scarcity in families? (b) How do families respond to resource scarcity? (c) What other factors influence family responses to resource scarcity?

## Method

### Data collection

To investigate family responses to resource scarcity, we conducted semi-structured interviews with 30 families living in the United Kingdom (UK). Similar to Epp and Velagaleti (2014), we focus on families with dependent children. Participants were selected using theoretical sampling (Eisenhardt & Graebner, 2007). Families were first approached using personal contacts, followed by snowballing, with the goal of achieving variability (see Table 3) on key criteria such as number of parents in the family, family type (traditional, single, blended, extended, same sex), family income, number of income earners, parent ethnicity, age, education level, occupation, country of origin, number of children in the family and their ages. Variability on demographic characteristics across respondents enables us to capture a wider range of potential responses to resource scarcity (Huberman & Miles, 1994).

**Table 3:** Summary of participant characteristics

	Name	No of Children	Children Age (years)	Family Type	Dual-Earner	Occupation	Parent Highest Education Level Attained	Family Income (£)	Ethnicity	Parent Age Group (years)
1	Marla & Nick^**^	2	4, 7	Two-parent, nuclear	Yes	M: SE, PT, Cleaner D: SE, FT, Cleaner	M & D: Professional Qualifications (Certificate)	26 – 50 K	White	M: 26–35 D: 36–45
2	Priya & Navin^**^	3	7, 9, 12	Two-parent, nuclear	No	M: Unemployed D: FT, Software Engineer	M: Undergraduate D: Postgraduate	51 – 75 K	Asian	D: 46–55 M: 36–45
3	Faith & Albert^**^	3	5, 9, 11	Two-parent, nuclear	Yes	M: PT, Finance D: SE, Taxi Driver	M: Undergraduate D: A-Level	51 – 75 K	White	D: 36–45 M: 36–45
4	Vicky & Robin	2	6, 9	Two-parent, nuclear	No	M: Unemployed D: FT, IT Consultancy	M: Undergraduate D: Undergraduate	> 100 K	White	D: 36–45 M: 36–45
5	Ellia^*^	2	11, 15	Single mum	No	M: FT Student, PT SME Business Developer, Church Volunteer	M: Postgraduate	26 – 50 K	Black	M: 36–45
6	Alan & Beth	1	7	Two-parent, living with extended family	Yes	M: PT Admin D: FT Sales Director, School PTA Lead	M: GCSE D: Professional Qualifications (Diploma)	51 – 75 K	White	D: 46–55 M: 46–55
7	Angelina & Max	2	8, 10	Two-parent, nuclear	Yes	M: PT Author D: FT, IT Consultancy	M: Postgraduate D: Postgraduate	76 – 100 K	White	D: 36–45 M: 36–45
8	Kate & Gabriel	4	9, 11, 16, 17	Two-parent, nuclear	Yes	M: PT Carer, Charity Volunteer D: FT, Bus Mechanic	M: A-Level D: A-Level	26 – 50 K	White	D: 36–45 M: 36–45
9	Eloise & Matt	2	8, 14	Two-parent, nuclear	Yes	M: FT, Nursery Teacher D: FT, Glazier	M & D: Professional Qualifications (Certificate)	26 – 50 K	White	D: 36–45 M: 36–45
10	Summer & Harry^**^	4	2, 5, 10, 14	Two-parent, nuclear	Yes	M: PT, Retail Customer Service D: FT, Civil Service	M: Diploma D: Postgraduate	51 – 75 K	Black	D: 46–55 M: 36–45
11	Jackie	3	6, 9, 9 (twins)	Single mum, divorced	No	M: PT, Medical Editor	M: Undergraduate	26 – 50 K	White	M: 36–45
12	Miranda & Alex^*^	2	11 months, 4	Two-parent, nuclear	Yes	M: SE, Admin D: Self-employed, Contractor	M: Undergraduate D: Undergraduate	76 – 100 K	White	D: 36–45 M: 36–45
13	Shruti & Ash^**^	2	5, 8	Two-parent, nuclear	No	M: Unemployed, Church Volunteer D: FT, Housekeeping	M: GCSE D: Professional Qualifications (Certificate)	0–25 K	Asian	D: 36–45 M: 36–45
14	Stephan & Esther	2	11, 14	Two-parent, nuclear	Yes	M: PT, Finance D: FT, Quality Assurance	M; Diploma D: A Level	51 – 75 K	White	D: 46–55 M: 46–55
15	Brenda	1	10	Single mum, divorced.	No	M: PT, Carer	M: GCSE	26 – 50 K	White	M: 46–55
16	Ana^*^	2	8, 19^a^	Single mum, separated, blended	No	M: PT, Finance	M: Professional Qualifications (Diploma)	0–25 K	White	M: 46–55
17	Anand & Reema	2	15 months, 2nd due	Two-parent, living with extended family	Yes	M: FT, Management Consultant D: FT, University Admissions	M: Postgraduate D: Undergraduate	76 – 100 K	Asian	D: 36–45 M: 36–45
18	Monique^*^	2	6, 12	Single mum, divorced	No	M: PT, Admin, Exports Business	M: GCSE	0–25 K	White	M: 46–55
19	Amelia & Jake	1	15 months	Two-parent, nuclear	Yes	M: PT, Chief Technology Officer D: FT, Chief Technology Officer	M: Postgraduate D: Undergraduate	76 – 100 K	White	D: 36–45 M: 36–45
20	Rose & Charlie	2	3, 7	Two-parent, nuclear	Yes	M: FT, Operations Manager D: FT, Financial Controller	M: Postgraduate D: Undergraduate	> 100 K	White	D: 36–45 M: 36–45
21	Ellie & Daniel^**^	2	4, 7	Two-parent, nuclear	No	M: Unemployed D: FT, IT Consultancy	M: Postgraduate D: Undergraduate	> 100 K	White	D: 36–45 M: 36–45
22	Ritika & Rishi^*^	2	7, 10	Two-parent, nuclear	No	M: Unemployed D: FT, Business Manager	M: Postgraduate D: Postgraduate	51 – 75 K	Asian	D: 36–45 M: 36–45
23	Candice	4	1, 8, 11, 14	Single mum divorced; blended	No	M: Unemployed	M: GCSE	0–25 K	White	M: 36–45
24	Louise & Jamie	4	5, 9, 10, 12	Two-parent blended	Yes	M: PT, Buyer D: FT, Director	M: A-Level D: Undergraduate	> 100 K	White	M: 26–35 D: 36–45
25	Lily^*^	1	18	Single mum	No	M: PT, Airline Duty Manager	M: Professional Qualifications (Diploma)	0–25 K	White	M: 36–45
26	Paul^*^	1	10	Single dad	No	D: FT, Construction Site Manager	D: A Level + Professional Qualifications	51 – 75 K	White	D: 36–45
27	Mark & Jack^*^	1	7	Same sex parents	Yes	Parent 1: FT, Company Director Parent 2: PT Teaching Assistant	Parent 1: Postgraduate Parent 2: Postgraduate	> 100 K	White	Parent 1: >55 Parent 2: 36–45
28	Julia & David^**^	2	5, 8	Two-parent, nuclear	Yes	M: FT, Software Developer D: FT, Designer	M: Postgraduate D: Undergraduate	Prefer Not to Say	M: Asian D: White	M: 36–45 D: 36–45
29	Ian & Eva^**^	2	8, 12	Two-parent, nuclear	Yes	M: PT, Carer D: FT, Engineer	M: Diploma D: A-Level	26 – 50 K	White	M: 36–45 D: 36–45
30	Vera & Tom^*^	1	13 months	Two-parent, nuclear	No	M: Unemployed D: FT, IT Consultancy	M: Undergraduate D: Undergraduate	26 – 50 K	M: White D: Mixed	M: 26–35 D: 46–55

D – Dad; M – Mum; FT – Full time; PT – Part time; SE – Self Employed; ^*^One of the parents born abroad, currently living and raising children in UK; ^**^Both parents born abroad, currently living and raising children in UK; ^a^ in full time education and still dependent on mum for most day to day expense; GCSE: General Certificate of Secondary Education: Awarded on successful exam completion after 5 years of compulsory secondary school; A-level: Awarded on successful exam completion after 2 extra years of non-compulsory secondary school

Parents served as informants for our interviews (e.g. Epp & Velagaleti 2014; Thomas & Epp, 2019), as they typically hold responsibility for generating resources and meeting the basic needs of the whole family. Families in our sample included both single-parent and two-parent households. In two-parent families, both parents participated in the interviews together. Involving both parents allowed mutual reflection and understanding of collective responses to scarce resources, through mutual sense making (Epp & Price, 2011).

Using McCracken’s (1988) funnel approach, the interviews began with fairly general questions about how many children were in each family, their ages, and how parent(s) managed household duties or chores, such as cooking, cleaning, child care and laundry. Subsequent questions prompted participants to recall specific previous experiences of time, space and money scarcity. For each type of resource scarcity, we explored whether families looked into alternative consumption options and if they considered investing more, less or similar amounts of the same or different resources. We also probed to understand how parents tried to balance their own utility and needs against that of other family members. Given the nature of resource scarcity, and the prevailing climate due to the COVID-19 pandemic at the time of data collection, participants were guided to form their narratives on scarcity not directly related to the pandemic except when prompted to do so.

We continued interviewing until the point of theoretical saturation (Creswell & Poth, 2018). A total of 30 families participated in our study. Table 3 summarises our participants’ profiles and uses pseudonyms to protect participant anonymity. Given restrictions in place due to Covid-19 at the time of data collection, interviews were conducted online using the Zoom platform. Interviews lasted between 45 and 110 min, with an average of 64 min, and generated a rich data set. Interviews were recorded, with participants’ signed consent, to ease the transcription process.

### Data analysis

Following theoretical thematic analysis procedures (Braun & Clarke, 2006; Raja et al., 2018; Bettany & Kerrane, 2018; Kerrane et al. 2021), data was analysed and coded at the family level. First, we familiarised ourselves with the data and extracted initial descriptive codes for each resource type for each family. Next, we collated the codes into broad themes to identify common strategies families utilise to respond to resource scarcity. We followed an iterative process, moving between our data, theory, and literature to uncover and refine emerging themes. We triangulated our data across resource types and families. Finally, the researchers re-grouped to define aggregate themes. Web Appendix 1 outlines the steps, and Appendix 1 presents the final coding structure derived from the data. Based on aggregate themes, we developed a conceptual framework of Family Responses to Resource Scarcity.

## Conceptual framework

**Fig. 1:**
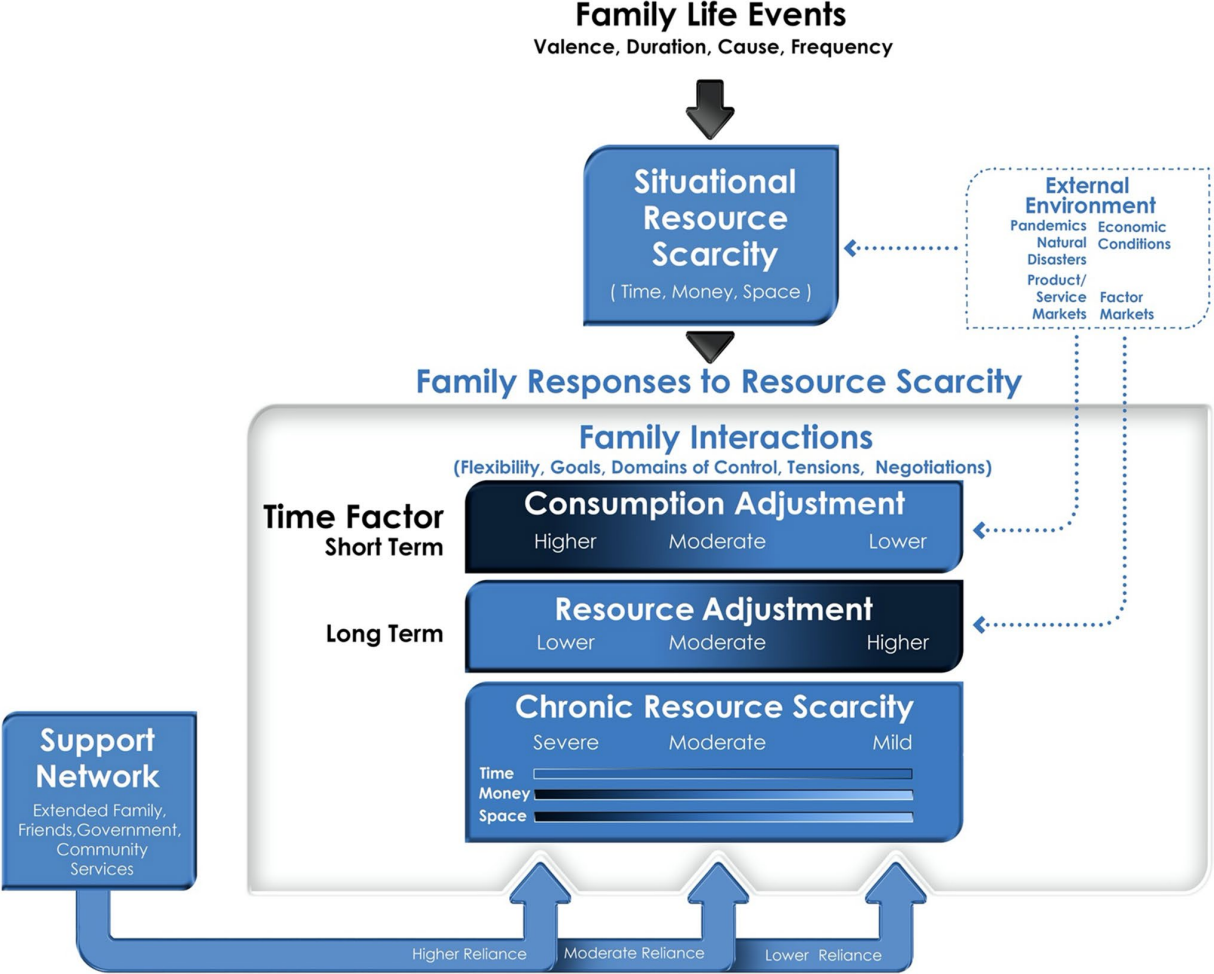
Conceptual framework of family responses to resource scarcity

Figure 1 illustrates how multi-dimensional family life events create situational resource scarcity. Families respond to situational resource scarcity by adjusting their consumption of goods and services in the short-term and resource investments in the longer term. These adjustments are influenced by family interactions, reliance on a support network, and level of chronic resource scarcity.

### Life events as triggers of family resource scarcity

Our interviews suggest that life events cause disruptions that trigger situational resource scarcity. Although scarcity can be induced by external events such as pandemics (Das et al., 2021), natural disasters (e.g. floods or fires due to climate change), economic (Hall, 2016) or political disruptions (Kerrane et al. 2021), we focus on how disruptions caused by life events, such as job transition, house move, child birth, wedding, divorce, diagnosis of major illnesses (e.g. cancer) or conditions (e.g. autism), prompt resource scarcity.

At the birth of a child, parents report that they spent time they would otherwise utilise to their own ends, for paid work, leisure or socialising, to cater to the new-born. Buying supplies, such a car seat, crib and changing table, requires reallocation of resources across family members and sharing (Belk, 2010). Consistent with Thomas and Epp (2019), childbirth as a life event creates a mismatch between expectations and actual requirements - perceived as resource scarcity. Full-time engineer Ian reflected on the space and money scarcity he and his partner confronted at the birth of their first child: “you don’t want to live in a shared flat [with friends], on top of the high street, [with a] shop downstairs, it was noisy … the thing we wanted to do [when our child was born] was to get out and move to a [more expensive] semi-detached house with a nice garden and still, not far away from where we’ve been before … [child birth] was obviously a life changing situation”. The family considered themselves fortunate to have their sister [extended family] living with them at the time to help with the caring duties of an autistic infant (time scarcity).

Single mum Jackie elaborates on how space for her family became scant after her divorce, given the reduction in household income, and her ability to borrow a lower mortgage [financial scarcity] (see also Thompson et al., 2018):

*… After the divorce, [we moved from a] ...5 to 3 bedrooms [house] … we’ve still got an open plan for the kitchen … but they [the children] don’t have as big a garden, [for] their climbing frame, or swing… I’ve got my bike set up as an indoor trainer … a lot of the time it’s here behind me ... [as] they’re [kids are] away for a few days [at their dad’s], it’s actually in the lounge, I wouldn’t do that when they’re here … [We now] share a bathroom, [which] we didn’t in the old place ... I get annoyed that the sink is covered in toothpaste and toothbrushes, whereas I used to just have my own …*

Eloise and Matt discuss the monetary resource scarcity they grappled with as they planned their wedding abroad:

*our wedding was difficult because we couldn’t have [all the] family … [we] couldn’t afford to pay for everybody … we tried to give people time … almost 18 months to two years notice - it wasn’t enough for some … it was quite sad … because I know that some of my family got themselves into debt on coming to our wedding … if I had the finances, I would have paid for people to go … .*

Although a positive event, the financial outlay required for the wedding was disruptive and caused Eloise and Matt to make other adjustments to their consumption. They saved considerably in the two years prior to the wedding, and arranged a local ceremony to accommodate their extended family and 7-year old god daughter on her death bed [time scarcity]:

*It was kind of a two thing [in addition to the cost of the wedding] … [god daughter] was dying [of cancer] at the time … it was the only time she was ever going to walk down the aisle … she was my bridesmaid ... [she] couldn’t [travel] because she was so ill … to compensate for Matt’s family not being able to be our wedding as well as give [god daughter] the day where she could wear white … it was all about making dreams come true for her* ...

The life events described by our respondents were sometimes due to voluntary decisions (such as Eloise and Matt’s wedding) and other times due to involuntary events, such as medical diagnoses. They may be of positive (e.g. childbirth), neutral (e.g. house move) or negative (e.g. divorce) valence, and can happen once (e.g. death) or multiple times (e.g. job transitions). Another dimension we used to categorise life events was duration: whether events generate transient or prolonged resource scarcity. For example, the initial diagnosis of a major illness can evoke immediate time scarcity. Over time, due to continued hospital visits for treatment and longer-term disruptions in income, the family adapts its consumption and resource generation activities. Table 4 depicts several life events as narrated by our participants along four dimensions.

**Table 4:** Dimensionality of life events

	Valence			Duration		Cause			Frequency	
	Positive	Neutral	Negative	Transient	Prolonged	Voluntary (Self Imposed)	Involuntary (Compelled)	One-Time		Recurrent
Pregnancy & childbirth	✓ (12, 17, 19, 23)			✓ (12,17, 19, 23)	✓^**^ All	✓ (12, 17, 19, 23)		✓ (19, 23)		✓ (12, 17)
Wedding	✓ (9)			✓ (9)		✓ (9)		✓ (9)		
Divorce		✓ (18, 26)	✓ (11, 15, 23, 25)	✓ (11, 15, 23, 25)	✓ (11, 15, 18, 23, 25, 26)	✓ (15, 18, 26)	✓ (11, 23, 25)	✓ (11, 15, 18, 23, 25, 26)		
Vacations	✓ (2, 3, 6, 7, 21, 24, 25)			✓ (2, 3, 6, 7, 21, 24, 25)		✓ (2, 3, 6, 7, 21, 24, 25)				✓ (2, 3, 6, 7, 21, 24, 25)
Hobby adoption	✓ (4, 7, 21, 25,28)			✓ (4, 7, 25)	✓ (21, 28)	✓ (4, 7, 21, 25,28)		✓ (4, 25)		✓ (7, 21, 28)
House move, purchase, or refurbishment	✓ (4, 7, 9, 17, 19, 20, 21, 28)	✓ (3, 14, 22)	✓ (2, 6, 11, 12, 23)	✓ (2, 7, 9, 12, 17, 19, 20, 21, 23)	✓ (3, 4, 6, 11, 14, 22, 28)	✓ (9, 14, 17, 19, 20, 22)	✓ (2, 3, 4, 6, 7, 11, 12 21, 23, 28)	✓ (2, 4, 6, 11, 12, 17, 20, 22, 23)		✓ (3, 7, 9, 14, 21, 28)
Secondary school transition	✓ (2, 4)			✓ (2, 4)		✓ (2, 4)		✓ (2, 4)		
Pet adoption	✓ (1, 4)				✓ (1, 4)	✓ (1, 4)		✓ (1, 4)		
Major illness / allergy Diagnosis			✓ (6, 9, 13, 17, 28, 29, 30)	✓ (9, 17, 30)	✓ (6, 13, 28, 29)		✓ (6, 9, 13, 17, 28, 29, 30)	✓ (9, 17, 30)		✓ (6, 13, 28, 29)
Death			✓ (9, 24)	✓ (9)	✓ (24)		✓ (9, 24)	✓ (9, 24)		
Job Transitions	✓ (4, 15, 19, 21, 27)	✓ (7, 8, 10, 18)	✓ (25, 26, 28)	✓ (4, 7, 10, 15, 18, 19, 21, 27)	✓ (8, 25, 26, 28)	✓ (8, 10, 15)	✓ (4, 7, 18, 19,21, 25, 26, 27, 28)	✓ (4, 8, 10, 19, 25, 26, 27)		✓ (7, 15, 18, 21, 28)
School breaks		✓ (9, 11, 13 14, 15, 16, 20, 28)	✓ (8, 23)	✓ (8, 9, 11, 13, 14, 15, 16, 20, 23, 28)			✓ (8, 9, 11, 13, 14, 15, 16, 20, 23, 28)			✓ (8, 9, 11, 13, 14, 15, 16, 20, 23, 28)

^**^Pregnancy & childbirth lead to childcare, which is resource intense, generating multiple forms of scarcity; Number in brackets denote participant family number

In Jackie’s case, divorce as a negative, involuntary, potentially one-time event (see also Thompson et al., 2018), can result in prolonged time, money and space scarcity. The family has to habituate to a smaller three-bedroom house (space) until the kids are old enough (time) to live separately or Jackie is able to afford a bigger property (money). In comparison, child birth is a positive, transient, voluntary, potentially recurring life event generates time, money and space scarcity for child care, over a prolonged period of time, given the resource intensity of parenting (Hays, 1996). Eloise and Matt’s wedding was a positive, voluntary, potentially one-time event that created scarcity over a more focused, transient time period. In this case, the family was confronting both financial (wedding costs) and time scarcity due to the deteriorating health of a close relative. For many of our informant families, one or more life events (e.g. wedding and life-threatening disease; home improvement and child birth) occurred simultaneously, generating multiple and concurrent forms of scarcity.

### Adjustment of consumption and resource investment

#### Consumption adjustment

Families respond to time, money and space scarcity by adjusting their total consumption of market supplied products and services. Navin and Priya live with their three boys in a rented apartment. The family intends to purchase a house with garden space for the children to play in the future. Saving for the mortgage deposit, together with paying monthly rent is strenuous. As Navin explains, “*….* we want to buy house but … [with] one person earning it’s a bit hard for us at the moment …” To save money, the family compromises on eating out, shopping, and limits their expenditures on entertainment. Single mum Ellia adjusts space consumption in her small, rented house by getting her teenage sons to be outdoors: “… you’ll see them [the boys] playing [sports] in the room. I’ll just say … go outside … there is parking space, but you need to be careful to play football, unless you go to the park”.

Families also respond to resource scarcity by reallocating or sharing (Belk, 2010) their consumption of products and services across family members (see also Web Appendix 2). In managing money as a scarce resource for her family, Marla prefers to sacrifice her own consumption on behalf of her family. Dad Ash manages scarce financial resources by relying on free school meals for his son’s lunch. Ash believes the school is very diligent in handling his son’s dietary requirements, allowing him to spread his limited budget across other members of his family: “the price [of food] is going up … my spend on food [is] a little less now … until the year 2s, [school is] helping [with] his lunches …”.

Ana explains how she moved furniture around the house to make space for her son: “… he asked me to buy a stationary bicycle … we have nowhere to put it, we struggle with space, I had to move the computer, dining table, store all my documents online …”. Mum of four children, Louise (blended family) emphasises the juggle across her family members over a busy weekend: “… we’ve got two [kids’] birthday parties on Saturday … and my sister’s graduation party … to fit that in, to make everyone happy is an absolute nightmare … Jamie’s [partner] going to drive to the parties … and I set up with the [other] kids for my sister’s party, and we meet up later on … it’s that juggling act …”.

#### Resource investment adjustment

In addition to consumption adjustment, families respond to resource scarcity through resource substitution and changes in resource investment (see Web Appendix 2). New mum Amelia recollects how she struggled with daily household chores at childbirth. As cooking three meals a day for everyone was time consuming, the family adjusted by purchasing ready-made food. Angelina, mum of two sons in a two-parent family, reflects on how she stepped up, adjusting her time investment, to handle a disruption in the family’s income: “at some point, Max had gone down to a four-day week, and his work was less certain. I’ve started to run online writing workshops to make a little bit of money ….” Illustrating resource substitution, families may spend money to acquire time (e.g. child care) or space (e.g. a larger house). Anand and Reema invested money in a bigger car to accommodate their growing family. Families also spend time to acquire money through the labour market in various work arrangements, often involving a mix of one or both parents in full time and/or part time employment. Mum of four, Summer, describes how she adjusted her permanent work schedule, and hence earnings, around child care: “I work Monday to Thursday. When I was looking for nursery for [youngest], they could [do] only four days … I work when all the [four] kids are at [nursery, primary and secondary] school …”.

Beyond substitutions of time and money, families utilise or sacrifice space to generate money. Single mum Monique elaborates on how she has been running her make-up exports business for years from home: “We’ve got a two-bedroom flat … my daughter got [her] room, my son got [his] room and I sleep in the living room … then there are the days when I pack it [make-up stock] into the big boxes and … my living room [bedroom] looks like a warehouse…”. Alan and Beth have lived with Beth’s parents for over eight years. With space of his own, Alan ascertains that he would gain time [minimise scarcity] and avoid hassle with his in-laws:

*[Every time] …. I have to drag everything [tools] out of the garage … if it rains, I’m … trying to put [everything back in] … if it were in our own place, … [when] I’ve had enough, [I would] shut the door on it … and it stays exactly like that* *…*

### Short-term versus long-term responses to resource scarcity

Looking back at our categorisation of life events as generating either transient or prolonged resource scarcity, we note that families tend to respond to short-term resource scarcity by adjusting their consumption of market bought goods and services. Parents postpone purchases or buy a cheaper alternative when money is tight, replace home parties with celebrations in restaurants when they are short of space, and compensate for lack of time to entertain children through purchases. In the short term, consistent with past research (Mullainathan & Shafir, 2013), family members tend to focus more attention on the scarce resource. During the process of responding to time constraints, and becoming more efficient (Fernbach et al., 2015), parents may give in to their children’s temptations for increased use of technology, thereby neglecting other relevant activities such as education or a healthy lifestyle. Resource scarcity imposes a cognitive ‘bandwidth tax,’ occupying the mind so much that limited resources are available for other activities (Mani et al., 2013).

Families with diverse demographic characteristics (see Table 3 - e.g. single parent, blended, and same-sex couple as well as traditional families) adjusted their total and per member consumption and resource investment over time (see Web Appendix 2). Single mum Brenda, explains how she grappled with budgetary constraints after her divorce. To make ends meet, she initially reduced consumption. Several years later, her daughter is at school and she can work part time to support her family:

…*We had a bit of a bad time … he [divorced husband] wasn’t paying any money at first … that was probably the worst time … I’m not one to ask my family … [for money] ... [I would] cut down on shopping and buy [less] … I would find cheaper stuff, look around … not spend too much…[Now] I work part time around the hours of school … I can [mostly] do the [work] hours around daily [matters], but if I couldn’t [my mum or my ex-husband are here for child care]* …

Similarly, Vicky and Robin from a well-off family, narrate how they adjusted consumption in the short-term, but invested in a bigger home in the longer run:

Vicky: *Robin’s parents came to stay [in our two-bedroom house], and we put them up in a hotel … we did have a sofa bed … but that’s not really welcoming …”.*

*Robin: “… we kind of put off inviting friends and family around … we thought it’s best waiting to have more space [move] ... Now that we moved, it’s a lot easier ….*

Over time, resource scarcity leads to gradual adaptation, learning and refinement of responses, so that families become more proficient in administering available resources (Dang et al., 2015). Parents are more likely respond to resource scarcity by substituting between, and changing their investment of resources across family members. A working parent may invest in more child-care, substituting money for time; a small home may be expanded; an unemployed parent may seek employment to generate more income. Overall, our observations converge across interviews (see Web Appendix 3), to suggest that family responses to resource scarcity evolve towards resource investment relative to consumption over time.

### Family interactions

Because families are made up of several members, they have more flexibility in adjusting their consumption of market goods and services in response to resource scarcity than individuals. Families portray flexibility in their responses to scarce resources, adjusting consumption of space by sharing bedrooms to make office space for the primary income earner and adjusting consumption of financial resources by passing hand me downs between siblings. Divorced mum of three, Jackie, highlights the challenges she faces in handling extra-curricular activities and holidays, as she shields her kids from the detrimental effects of budgetary constraints. She embraces flexibility by reallocating scarce finances across her three children:

Jackie: *All three of them [don’t] have swimming lessons, because it’s very expensive ... so the two that can swim [twins, 9 years old] don’t have lessons anymore ... I take them to [free/uncoached] swimming, while the other one [younger, 6 years old] was having a lesson…that was a sort of an adaptation, you know, I can’t do swimming lessons for all of you, but I’ll take care of your swimming …*

Researcher: *Was it difficult /challenging to handle that?*

Jackie: *I suppose I didn’t make it explicit that they [twins] are having to miss out when somebody else [younger brother] is doing something, I try and do that subtly … they’re not aware that I’m not taking them swimming, because I can’t … [Thinking] back to the [kids] holidays as another example, they’re aware that we can’t necessarily go on a on a big holiday … actually, their dad quite often takes them somewhere, so they don’t exactly miss out. They just miss out on it with me … because there’s not enough spare money for the holidays, we go camping or we do fairly low-key holidays …*

Flexibility within the family is also enacted by substituting products and services for scarce resources. Resource scarcity broadens considerations sets and stimulates consumers to be creative (Hill et al., 1998). Ellie and Daniel’s old house was so small for their family of four that they had to be out most weekends: “… [due to lack of space], on Saturday [we] would go to soft play … then on Sunday, we could take them shopping, [and] even buy some presents [toys] for them …”. Instead of spending on larger living quarters in the short-term, the parents utilised products and services to respond to space scarcity, as they saved up for a bigger house.

Our interviews suggest that collective goals provide the over-riding basis for parents’ allocation of scarce resources on behalf of their families, consistent with earlier research on family consumption (Epp & Price, 2011). Families stress the significance of managing within budgets and saving as a goal in responding to financial scarcity. Single mum Candice discusses her profound sense of responsibility and tension in pursuing the family goal of administering a tight budget:

*My kids constantly need new clothes, because obviously they’re getting bigger … so I sacrifice my clothing for my children … I’ve got clothes that I’m wearing that I wore five years ago, because … I would rather my kids have new clothes, and shoes and things … if they need a haircut, I love [prefer] the £10 to go on their haircut, than £20 on mine … so I neglect my hair … I sacrifice a lot …*

Julia and David, draw on the challenges and responsibilities they confront as parents of an 8-year old autistic and disabled child, to secure family welfare:

Julia: *People think it’s difficult being without holidays … and they suffer …. [for us] …. like in school holidays, we cannot switch off … we have to be hands’ on for him [disabled child] …. that’s the reality …*.

David: *If you have children, [you] dedicate to this … you surrender your schedule ….*

Julia: *We [can] go to like four or five days but we can’t go for longer … because there’s no respite [charity/child support] longer than a week … even with the respite …we have to plan ahead and [still] it can get cancelled last minute and all the planning goes down the drain … The only things we can do is watch Netflix or stay local …*.

Beyond family goals, tensions, challenges and flexibilities, individual family members maintain control or authority (Epp & Velagaleti, 2014) over certain domains in their responses to resource scarcity. Kate considers that, as the mum of a dual-earner family, she has more responsibility than her husband for handling her four children’s daily needs and requirements. She feels that she knows best how to appropriate the family budget:

*Generally, every time we get paid, we discuss what is going on, whose wages [will go to] rent, council tax, which are always the biggest things ... and then it’s food, … and I work out what’s left for everything else … As the mum, I think I’ve got to manage the kids more … I have to work it out [as] I know what works really … whatever I say goes mainly for final decisions ….*

The inter-relation between collective goals, integral flexibilities and tensions due to the presence of several family members, and adherence to domains of control, results in complex family negotiations (Spiro, 1983; Qualls, 1988; see also Web Appendix 4), determining family responses to resource scarcity. Time-stretched, Priya and Navin build consensus within their relational unit as mum and dad (Epp & Price, 2008), then negotiate with their kids for them to complete homework:

Researcher: *Who has more control over the kids’ education?*

Navin: *It is a matter of mutual agreement between two of us [parents]*….

Researcher: *How do the kids feel about that?*

Priya:* They sometimes hate, sometimes love, sometimes cry, … we sometimes bribe them, sometimes get angry ... it’s a mixture of things* ...

Our findings further reveal how families grapple with resource scarcity by embracing flexibility in adjusting their total resource investment across family members. When Marla’s husband is busy with work, she will assist him in running the family business, in addition to accomplishing household chores: “I do all things at home. I went to buy something for him [husband] to help him out … for his job, … like collecting his online orders of screws or chemicals …”, thus invoking adjustment of resource investment across family members and flexibility. Same sex parents Mark and Jack portray flexibility in substituting space and money as they permanently altered their house set up to work from home and accomplish both their goals of being productive and caring for their son:

Mark: *The [current] office was just [cluttered with] clothes, etc… for a long time … so Jack sort of kitted that out … [it is] now is a very productive space*.

Jack: *[I] cleared out, tried to push some cushions on the floor, some blankets, and did some other work to it … I said to [son] look, you can play here with your toys or games or even your tablet [and you] can ask me any questions ... he is very happy …*

Mark:* We’re also lucky that we work quite differently … like Jack [can] have the office because [he] can close the door and just stay in there for six hours. But I like to work on the kitchen table because I like the noise and distraction and he doesn’t …*.

In handling adjustments in resource investment across family members, we note the relevance of collective goals (Epp & Price, 2011), domains of control (Epp & Velagaleti, 2014), and negotiations (Spiro, 1983; Qualls, 1988; see also Web Appendix 4). Dad Alex explains how kids’ toys have dominated their small family home over the last four years. Alex expresses frustration that, within the clutter, he does not have an office space and has to invest in garages to store his work tools and spare parts. Julia recalls the challenges her family had to grapple with as they renovated their home to accommodate their disabled son, investing time and money to secure well-being as a family goal:

*We [adapted the house] … because [it has] to be a welcoming hospital … to feel like a home [with] a lot of specialised equipment … [designer dad, David] planned the space … we didn’t have a bathroom downstairs … [it was] incredibly difficult because you got to carry him [son] up and [down] … we had a Victorian house [with steep stairs] … we remodelled the house, [adjusted the] staircase, added a bathroom and bedroom for him downstairs so he doesn’t need to go up … we have a really, really good neighbour … he rented his house next door to us for nearly five months [during the extension] … it was stressful … it took us nearly two years to finish … [the new] roof was leaking [in the winter] … we adapted the whole house so that the wheelchair can go around … we extended the house sideways and into the garden …to create a bright room for [son] … the council gave us £30K grant … which is, [similar to the grant of] 20 years ago … the total cost is about £200K and we had to re-mortgage and use all our savings ….*

When making decisions about resource substitution and investment of resources by various members, families are also guided by domains of control. One parent may be better skilled at, or used to doing, a given task, thus giving them more control in that area when resource scarcity arises. Alan recounts how he makes most financial decisions, bringing his wife in on an as needed basis: “Generally, all financial decisions, I deal with it, but obviously, [if] there was something major, that’s a joint discussion, but for the day to day running [of] the family, I just get on and do whatever we’ve got to do …”. In Eloise and Matt’s family, Eloise prefers to leave investment decisions regarding space to dad Matt, given his building skills, even if she proposes the ideas:

Eloise: *But when it comes to working out [spaces], I wouldn’t [interfere]* ….

Matt: *You suggest the ideas* ….

In other families, decision making is more balanced, with both partners sharing responsibility. In the case of Rose and Charlie, dad Charlie researches resource investment decisions with high financial outlays, such as nursery for their boys (time) or contractors for their home extension (space). Information gathering is followed by conversation between the two parents, with ultimate choice depending on matters such as domain of decision making and level of expertise and motivation:

Rose: *You [dad, Charlie] did the research and then we probably [discuss] …*.

*Charlie: It depends on which area of decision making [it is], who might have more say, or feels most passionately, or is most affected by it …*.

*Rose:* *I will probably choose the nursery and Charlie the contractors … but … [we are] fairly diplomatic ….*

In dealing with limited finances, Angelina and Max embrace a soft bargaining approach, as they convince their kids to save money: “We look for more win-win … what’s kind of best for everybody, the way we say No to things [with kids] is we explain why … it’s very much kind of talk them into submission … [saving their pocket and birthday monies].”

### Reliance on support network

Our findings (Web Appendix 5) highlight the role of help from the extended family, friends and neighbours (also referred to as the resource mix by Epp & Velagaleti 2014), as families respond to resource scarcity. Beth and Alan share a house with their parents/in laws and receive regular help with child care. Miranda and Alex have occasional support from Alex’s mum for child care as she lives significantly further away. Single mum Brenda has considerable support from her own mum as the family lives within commuting distance. Nick and Marla, who are from abroad, but living and raising their children in the UK, receive child care support only when they go on holidays to visit their extended family.

In addition to time, financial resources may be shared across family members. Single mum Ellia sometimes receives monetary help from her extended family from abroad. Monique obtains financial support from her divorced husband, who lives an hour drive away. Families also rely upon non-family support networks when they did not have extended family nearby. Lisa details the support she received to grapple with financial scarcity as her household income halved when her partner left:

*Since [my ex-partner] left … there was a decrease in income … I was really worried … and I said to him[son]: Listen, we’re gonna have to make sacrifices … My landlords were very understanding … they reduced my rent and I attended a financial planning course offered by the church …*

### Chronic resource scarcity

We further note differences in responses to situational resource scarcity based on families’ levels of chronic resource scarcity. In Table 5, we compare responses by families encountering severe, moderate and mild chronic resource scarcity.

**Table 5:** Comparison of family responses by resource levels

	Severe Chronic Resource Scarcity Typically: ▪ Low income earners £ (0–25 K), or low income per family member ▪ Basic level of education ▪ Single parent or one income earner ▪ Low skilled jobs: unemployed, cleaner, caregiver, mechanic, admin	Moderate Chronic Resource Scarcity Typically: ▪ Mid income earners £ (26 – 75 K), or medium income per member ▪ Mix of education levels ▪ Mix of one or two-income earners ▪ Mid skill jobs: engineer, teacher, designer	Mild Chronic Resource Scarcity Typically: ▪ High income earners £ (> 76 K), or high income per member ▪ Higher level of education ▪ Two parent families ▪ Professionals: consultants, business owner, company director, lawyer
Time Scarcity	Child care; daily activities, personal time	Child care, education, personal time	Child care, hobbies, personal time
Response	Higher consumption adjustment: use of paid facilities e.g. play-centres; technology Lower (e.g. part-time work) to practically no resource investment (e.g. paid child care)	Mix of consumption adjustment: use of paid facilities (after school clubs, tuition, play centres); and resource investment (part-time work, work from home) and child care options (e.g. child minders)	Higher resource investment: full time nursery, au pair Lower consumption adjustment: use of paid facilities e.g. ready meals, technology
Money Scarcity	Essentials: rent, daily meals, school dinners, clothing, day to day maintenance e.g. haircuts	Non-essentials: pets, gifts, leisure, gaming, holidays, home refurbishment and purchase	Luxuries: holiday, home and garden refurbishment, bigger house purchase, private schooling
Response	Higher consumption adjustment: constant savings, buying on deals, budgeting, prioritise expenses, use / purchase second hand, local entertainment, smaller houses Lower resource investment: additional (often part-time) employment, supplement with elder kids’ part-time salaries	Mix of consumption adjustment: savings and budgeting for big purchases, buying on deals, low key holidays, scale down home improvements, lifestyles; and, resource investment: additional employment (usually other partner), utilise alternative financing options	Higher resource investment: additional employment, rely on bonus, relocating Lower consumption adjustment: fewer trips abroad, local theme parks rather than travelling
Space Scarcity	Small houses and outdoor spaces	Outdoor play/garden, storage, office space	Office space, handling hobbies and guests
Response	Higher consumption adjustment: share bedrooms, staying with family, utilise communal play areas Little to practically no resource investment	Mix of consumption adjustment: share bedrooms, technology use, local activities, outside storage, stay with friends/family; and resource investment: home improvement	Higher resource investment: hotels to accommodate guests, house moves, refurbishment, relocation Little consumption adjustment
Support Network	High Reliance	Medium Reliance	Low Reliance
Family Participant No.	1, 8, 13, 15, 16, 18, 23, 25	2, 3, 5, 6, 9, 10, 11, 12, 14, 22, 26, 28, 29, 30	4, 7, 17, 19, 20, 21, 24, 27

#### Severe chronic resource scarcity

As illustrated in Table 5, families with the lowest level of resources face severe chronic resource scarcity. They tend to have a basic level of education, usually with only one parent earning income, in low skilled jobs such as cleaners, mechanics, caregivers; or even unemployed. Such families struggle for daily essentials, such as rent, food, clothing, and face more space constraints. Prior to receiving government housing (support network), single parent Candice (family 23) had to share a room with a double bunk with her kids, in her disabled parents’ house. Sharing space (consumption adjustment) allowed the family to temporarily get by on a lower level of resources. Although she was constrained to work only during school hours, Candice adjusted her resource investment (time) in the labour market outside of regular schedule, when pregnant with her fourth child, with some help from her parents to get her kids ready for school (support network).

#### Moderate chronic resource scarcity

Families enduring moderate chronic resource scarcity typically earn higher incomes, are more likely to live in two-parent families and work in skilled jobs. Like families with severe chronic resource scarcity, moderate resource families report time constraints to balance childcare, with paid employment. Although such families can afford the basics, they were still restricted on non-essentials such as pets, leisure activities, and gifts. To cope with financial scarcity, informants indicated timing purchases to buy on deals, saving and budgeting. Space scarcity was more often reported for storage and activities such as exercise or home office, although they still described children sharing bedrooms (consumption adjustment). In other cases, families invested financial resources via home improvements to respond to space scarcity.

#### Mild chronic resource scarcity

Families with the highest level of resources face only mild chronic scarcity. They tend to have higher family incomes, with both parents having attained a higher education level, and one or both parents employed as professionals. Like other families, mild chronic resource families report time scarcity, particularly for child care. For instance, Louise (family 24), a mum of four children from a blended family, describes feeling short of time, but hires an au pair to help with the children and can afford to reduce her work hours when needed (shifts in resource investment). Monetary scarcity is endured by families facing mild chronic resource scarcity for luxuries, such as expensive holidays and private schooling. Space scarcity is encountered only in reference to practising hobbies and accommodating guests. Louise has a big open kitchen and dining space, each of her four kids have their own bedrooms, the family has home offices for the parents, an outhouse and a boat.

As per Table 5, families with differing levels of chronic scarcity all note time constraints, but face money and space scarcity to various degrees. Notably, families at all three levels of chronic resource scarcity describe shifting consumption of products and services, and investment of resources, but to contrasting levels and rhythms. Thus, childbirth as an intra-family life-event, creates situational resource scarcity, requiring extra time, money and space. In responding to situational resource scarcity, severe chronic resource scarcity families, are more likely to accommodate the new arrival through consumption adjustment. The family may rely on hand me downs (money scarcity), sharing bedrooms (space scarcity), better planning and prioritising of family roles and responsibilities (time scarcity). With time, the family may (if at all) invest in a bigger home (resource investment). A family facing mild chronic resource scarcity may in the short-term rely on ready-made food (consumption adjustment), as they hire a nanny and upsize to coincide with child-birth and minimise resource scarcity.

We also observe greater reliance of families facing higher levels of chronic resource scarcity on government, church or the community, as they cannot afford paid support. Single mum Ellia, relies on support from the community for her sons to practise sports: “[The boys] like football … and they [the coaches] usually come and pick them up …. because I don’t drive …. [and there is no one else to take them]”. In contrast, Rose and Charlie, both in full time jobs with a high family income, outsource many tasks, and rely on some support from their extended family, but not from government or community members:

*We spend more, ... the nursery ...is probably more expensive [due to] longer hours, we get take away … we don’t have time to cook ... we have a cleaner … But, you choose to work ... you also earn more to compensate … my mum used to come every week, and help … she would do laundry and stuff for us ….*

## General discussion

### Theoretical implications

#### Family responses versus individual responses to scarcity

In extending research from individual to family responses to resource scarcity, we note the relevance of family interactions comprising of goals, flexibility, tensions, domains of control and negotiations. To his end, family members share resources, adapt consumption and resource investment in flexible ways, often unmatched by individuals. When individuals face resource scarcities along their consumer decision journeys (Hamilton et al., 2019b), they can set priorities, engage in planning (Shah et al., 2012), delay gratification (Mittal & Griskevicius, 2014), increase the size of their consideration sets and use substitutes (Hill et al., 1998).

The additional flexibilities conferred by multiple members allow families to respond in different ways from individuals. Family members can adjust and share consumption of products and services. In addition to substituting between resources, as individual consumers, family members can change the way their members invest resources. Parents, and in some cases, children, substitute for each other’s time, money and space as they utilise resources to achieve collective goals. Despite the greater flexibility of family members, conflicting individual and relational priorities (Epp & Price, 2011) often result in tensions, challenges and negotiations across family members. Whereas an individual can delay personal consumption or substitute between resources, relationships and responsibilities contribute to the tensions and burden of responding to resource scarcity in families.

Parents play a dominant role in making challenging consumption and resource investment decisions due to scarcity. Although they typically have higher power and responsibility (e.g. Childers & Rao 1992) due to their role in resource generation, parents and children adopt a range of negotiation or bargaining tactics (Isler et al., 1987; Cowan et al., 1984) for resource allocation and enhanced family value (Becker, 1965). Older children can generate resources working part time to satisfy their or their families’ consumption goals; they can also look after younger siblings - usually at the command of their parent(s), rather than volunteering. Children also claim a greater influence on scarce resources due to their developmental needs, even if, they need to be taught to share, with sharing, allocation and use of resources, not necessarily being impartial (Belk, 2010). In many cases, mothers sacrifice on behalf of their families, as the strain of careers, invisible, physical and/or emotional household labour rests on them (Hothschild, 1989). Despite shifting gender norms, “A mother’s work remains never done” (Ciciolla & Luthar, 2019: 468).

Across structures, we observe that family members engage in negotiation and experience conflict as they decide how to adjust consumption and resource investment in response to resource scarcity. Family members embrace specific domains of control based on their expertise or roles within the family. In some of our participant families, one parent considered themselves better suited to allocate resources to their children, assuming that they knew the children, their routines, and critical needs better. In line with bargaining theories of family decision making (Blood & Wolfe, 1960; French & Raven, 1959), we find that family members who believe they have more authority, expertise, or knowledge maintain higher control over specific domains of activity and are more likely to guide the family’s responses. Consistent with Relative Investment Theory (Davis, 1976), family members motivated to get their way, also have stronger influence on responses to resource scarcity. Thus, relationships and interactions play a significant role in family responses to resource scarcity.

#### The role of life events in resource scarcity

Our research notes a range of intra-familial, scarcity inducing life events that occur along the life course, from birth, through school transitions, sickness to death. We also identify several dimensions of life events that generate situational resource scarcity, including valence, duration, cause and frequency. Notably, a single life event may create multiple forms of resource scarcity. Time, money and space scarcity often accompany childbirth or medical diagnoses. Life events may also occur simultaneously or in succession. After a family welcomes a new child, requiring significant money, time and space, one parent may leave their job, further constraining finances. When multiple and concurrent life events occur, goals and priorities become pertinent as families respond to resource demands.

For example, if one family member is inflicted with a major illness, the initial focus will be on treating and restoring his/her health. However, the resource demands of other family members (e.g. child care) and life events (e.g. job transitions) remain. In the immediate term, the needs of other family members may be met through consumption adjustment such as meal deliveries or household chores outsourcing, depending on the family’s chronic resource scarcity. Flexibility may be enacted through the help of another parent (two parent family), friends or extended family, acting as a support network. If the illness persists, the family may undertake larger resource investments as they develop a longer-term care plan. Such decisions will generate tensions, challenges and discussion on domains of control, as the family habituates to the disruption of this medical life event.

Contrary to previous resource scarcity studies examining responses at the individual level (Table 1), and family consumption research investigating relatively distinct life events (Table 2), we contribute to both literatures by integrating our examination of multiple life events in families with dependent children, through the theoretical lens of resource scarcity. In studying the resource implications of life events that happen concurrently and sequentially, we observe that families’ consumption orientations are geared towards flexibility, adaptability and fluidity, corresponding to the dimensions of liquid consumption (Bardhi & Eckhardt, 2017).

#### Responding to resource scarcity over time

Another notable insight we draw from our empirical work is the temporal nature of family responses to resource scarcity. We build on the theorisations of Fernbach et al. (2015) and Mullainathan and Shafir (2013) in proposing that families, similar to individuals, aim for efficiency in responding to resource scarcity. Such efficiencies stretch further in family units, with evidence of consumption being adjusted across several family members. To maximise utility, families experiencing a shortage of space adjust in the short term by sharing space. One parent may use the home office during the day, and the other in evening, or one parent may occupy the kitchen or dining table while the other uses the home office. In the short term, families may respond to space scarcity by purchasing a product that allows them to use existing space more efficiently, such as a new desk.

In the longer run, families respond by substituting between the resources they invest. Our study reveals how families substitute between time, money and space to increase utility (Becker, 1965). A family short of time for child care will invest in day care services; another family with budgetary constraints will substitute their time for paid work. A family experiencing scarce space may substitute money for space by extending their house to add a new office. A family experiencing money scarcity for holidays abroad may, in the short term, adjust consumption by going to local theme parks. In the longer run, the same family may engage in extra paid work, substituting time for money, to enjoy a holiday abroad.

In theorising the temporal nature of family responses to resource scarcity, we also note this reasoning fits Hamilton et al.’s (2019a) conceptualisation of consumer responses to financial constraints. Hamilton et al. (2019a) propose that consumers manage financial scarcity through a series of sequential steps, where they react and cope (adjust) in the immediate term, and then adapt, changing the way they respond (invest) in the longer term. In effect, our findings extend the temporal nature of responses to scarcity beyond financial constraints, to examine responses to space and time resource scarcity.

Across the resources of time, money and space, we reflect on how families as consumer units, navigate collective consumer journeys in their daily and long-term pursuits (Hamilton & Price, 2019; Thomas et al., 2020). As much as family goals provide the over-riding structure for the prioritisation, allocation, substitution and consumption of resources, situational constraints from life events often dictate how family members trade-off between resources at various points of the consumer journey. Consistent with Household Production Theory (Becker, 1965), family members combine market bought products and services with their own resources for utility optimisation. Thus, in addition to substitution among resources, families may also act as resource integrators (Hamilton & Price, 2019), where combinations such as time and money, or money and space are combined to achieve life objectives.

#### Interaction between situational and chronic resource scarcity

Our work further recognises how family responses to situational resource scarcity differ based on chronic resource scarcity. Irrespective of severe or mild chronic resource scarcity (see Table 5), almost all families experience time scarcity, primarily related to child care. In response, families adjust their consumption of market supplied goods and services (e.g. use of technology or play centres) and their investment or substitution of time into the labour market for money. In some families, one parent stayed home to look after the children or worked part-time hours, thus sacrificing earnings. Families facing mild chronic resource scarcity were more likely to invest in expensive nurseries to gain time, and invest in the labour market. Families facing moderate chronic resource scarcity relied on government funded nurseries (support network) to join the labour market after their kids reached a certain age. Families confronting severe chronic resource scarcity benefitted more from support networks, relying on extended families, the community and government for child care.

Families’ chronic resource levels also led them to respond differently to money and space scarcity. Families grappling with severe chronic resource scarcity are more likely to respond through higher consumption adjustment; families enduring mild chronic resource scarcity through higher resource investment, while families in between with a roughly equal mix of each (see Table 5). Initially, most families respond through some form of initial consumption adjustment and proceed to resource investment. However, the speed with which families switch to resource investment differs based on the level of chronic resource scarcity. Through their financial and cognitive resources (e.g. education level), mild chronic resource scarcity families move to a permanent solution to their resource constraints faster.

By overlaying the chronic resource scarcity (severe, moderate, mild) with the situational scarcity encountered by families through life events, this study responds to Hamilton et al. (2019a) and Goldsmith et al. (2020a) calls for future research. Hamilton et al. (2019a) call for future research to examine whether the interaction between chronic and situational resource scarcity leads to consumer reaction, coping or adaptation. In this context, and as we summarise in Table 5; Fig. 1, families respond through a mix of consumption adjustment (react and cope), resource investment (adaptation), and reliance on a support network. Our study also responds to Goldsmith et al.’s (2020a) suggestion that responses to resource scarcity derive from situational and chronic conditions. Table 6 compares our contributions relative to existing literature on resource scarcity and family consumption.

**Table 6:** Summary of contributions

Contributions	Related Ideas from Past Literature	Novel Contributions
Resource Scarcity at the Family Level	▪ Families are an important consumer unit with individual, relational and collective goals (Epp & Price, 2008, 2011). ▪ Environment trends create shifts in traditional, parenting roles and generate flexibility between family members (Epp & Price, 2018). ▪ Due to individual family member motivations (Davis, 1976), negotiations are an important aspect of family decision-making (French & Raven, 1959; Blood & Wolfe, 1960). ▪ Individual responses to resource scarcity promote behaviours like selfishness (Roux et al., 2015; Goldsmith et al., 2018).	▪ Building on research at the individual level, we investigate resource scarcity at the family level. ▪ Family responses promote flexibility, sharing (Belk, 2010), negotiations (Cowan et al., 1984), interactions and tensions due to individual, relational and collective goals (Epp & Price, 2008, 2011). ▪ In a developed economy, parents tend to be the primary resource generators and purchasers, assuming responsibility for meeting the basic needs of all family members. Parents feel tension, undergo negotiations, given domains of control and their responsibility in fulfilling collective goals.
Life Events	▪ Existing research independently investigates how one-time, or costly events, such as child birth (Thomas & Epp, 2019), family vacations (Epp & Price, 2011), child care (Epp & Velagaleti, 2014), or mothers ‘prepping’ behaviours (extra food stocking) in anticipation of UK Brexit (Kerrane et al. 2021) lead to changes in family resource allocation.	▪ Scarcity inducing life events can be categorised on: valence (positive, negative or neutral), duration (transient or prolonged), cause (voluntary or compelled) and frequency (one-time or recurrent). ▪ One of the few empirical attempts to investigate how multiple life events, concurrently and consecutively, create one or more forms of resource scarcity.
Time Factor	▪ Individuals handle money scarcity through adjustment in the short term, and investment in the longer term (Hamilton et al., 2019a).	▪ This research extends Hamilton et al.’s (2019a) theorisation beyond finances to time and space scarcity. ▪ Families substitute between time, money and space. ▪ Family responses involve adjustments to: (i) consumption in the short term, and, (ii) resource investment in the long term.
Interaction between Situational and Chronic Scarcity	▪ Studies independently investigate individual responses to situational or chronic scarcity (see Table 1).	▪ Family life events inflict situational resource scarcity. ▪ Families face severe, moderate or mild chronic resource scarcity. ▪ Families respond to resource constraints depending on their structures, through a mix of consumption adjustment, resource investment and reliance on their support network. ▪ Responds to the call for future research by Hamilton et al. (2019a) and Goldsmith et al. (2020a) by examining the interaction between chronic and situational resource scarcity.

Reflecting on our conceptual framework (Fig. 1), we observe how Covid-19 as a ‘black swan’ life event (external environment), with widespread economic, health, emotional, psychological and societal consequences (Taleb, 2008), prompted families to respond to resource scarcity. Initially, families responded to time and space scarcity mostly by adjusting their consumption of arts and crafts, garden equipment and streaming services. Kitchen and dining tables were utilised for school and office work. We also observe evolving responses to scarcity. With the easing of social distancing, time scarcity was reduced by sharing child care responsibilities across parents (e.g. divorced), support networks and childcare providers. Gradually, families also engaged in home improvements or upsized (resource investment). Families facing mild chronic resource scarcity swiftly adjusted resource investment to minimise constraints (e.g. home refurbishment). Severe chronic resource scarcity families relied more on consumption adjustment and more gradually moved to resource investment (if at all). Overall, flexibility and domains of control within families influenced responses, as parents negotiate tensions to uphold collective goals.

### Practical implications

At a practical level, our research offers managers insight into how families adjust their consumption and substitute between resources to respond to resource scarcity. For example, the need for home space organizers, such as toy storage boxes, becomes more obvious to the marketer, when he/she understands that parents face space constraints. Similarly, regular visits to soft play centres, or sports clubs, become more explicit to businesses, when they realise that they are providing an outlet for families to be away from their over-crowded homes.

#### Tailoring marketing activities to life events

The multitude, concurrence, simultaneity and multi-dimensionality of life events, provide multiple opportunities for tailoring marketing activities. Businesses need to recognise the resource implications of life events on families. While death is negative and entails mourning (Whitley et al., 2021), a wedding is positive and celebratory. Across differences in the valence, cause or frequency of life events, consumption enables a form of control (Pavia & Mason, 2004) for consumers. Family members seek to construct new identities following situational scarcity induced by life events. Families downsize, live on a lower income and use public transportation after divorce (Thompson et al., 2018); engage in new routines after childbirth (Thomas & Epp, 2019) and outsource child care (Epp & Velagaleti, 2014). Holidays and gift-giving acknowledge the presence of new life partners (Otnes et al., 1994).

Businesses therefore need to be on the look-out for families experiencing life changing events, develop relevant segmentation and targeting, based on the level of chronic resource scarcity, to build their customer base. Marketers may consider introducing multiple priced versions of time, space and money saving innovations, to cater for different types of resource scarce families undergoing life events/transitions. Marketers should also be aware of the frequency of life events as families change their consumption habits to ease transitions and embrace change. One-time events such as a first child, wedding or death may reduce variety seeking and price consciousness in purchases, compared to recurrent ones (e.g. second child, adoption of hobbies; Koschate-Fisher et al. 2018).

#### Technology solutions

Access-based providers of products and services, enabled by the digital revolution and sharing economy (Eckhardt et al., 2019) should target families with children facing time scarcity. The provision of one to one, online support to help children with their homework, or perform extra-curricular activities, via Zoom or Google Meet, eliminates the need for parents to spend time travelling to bridge space, for physical class attendance. Supermarkets may partner with delivery apps to boost capacity and ease parents’ time scarcity. Targeting families for meal deliveries through services like Uber Eats, or partly prepared options such as Simply Cook, to alleviate time constraints, may convince families facing time scarcity to substitute home-cooked meals. Online shopping, socialising, health and fitness apps all provide avenues for businesses to tap into and assist families in managing scarce time.

Technology also provides solutions for money-stretched families. Facilitating exchanges between families with too many belongings, or too much food, but little space and/or facing monetary scarcity will make both better off. Families can utilise sharing apps such as OLIO to bid for and accept donations of food items close to their use-by dates, from well renowned food stores, or people within the community. Supermarkets may also launch pre-made food parcels at multiple price points targeting families facing time or money constraints. Apps could also be utilised to facilitate family time commitments as they juggle work, school, extra-curricular activities, shopping, cleaners and general household tasks ranging from birthday planning to home improvements. Apps such as OurGroceries or Google Calendar could act as a communication tool to maintain family relationships and ease time scarcity.

Given the recent boost in working from home due to Covid-19, our findings may be useful to employers revising their policies. Working from home promotes work life balance and may alleviate chronic time scarcity for families with children. However, new ways of working may induce space scarcity as employees need more equipment at home. Given the significant reduction in commuting time and costs and the associated reduction in carbon emissions, employees may be willing to sacrifice space in the short term for increased home office requirements. In the longer term, digital/remote work places may promote relocation to mitigate multiple forms of scarcity concurrently. Family flexibilities, substitution between resources and the critical relevance of geographical proximity to extended families as one component of families’ support network, may allow parents to utilise the resulting extra time to handle other daily duties or enhance general well-being.

#### Enabling resource substitutions

Resource substitutions may be illustrated via story-telling in advertising, to go beyond product use and create strong emotional connections with consumers (Woodside et al., 2008). Stories depicting life events, their accompanying resource scarcities and solutions in a narrative way, can elicit long term intentions and attitudes (Van Laer et al., 2014). Understanding family resource substitution will also enable businesses to handle some of the grand challenges, such as sustainability, facing society. Our research uncovers how consumers facing financial and space constraints are inclined to engage in physical or online reselling of their used items. Families are able to dispose of their unwanted belongings, free up space, procure money and eventually acquire possessions of choice, if any, at a cheaper price within the same outlets. We therefore advocate the need for businesses to be on the look-out for innovative resource to resource substitution in consumer lives, and set up the right physical or online platforms to target their offerings or enable consumer to consumer exchanges.

Equally, we contend that the provision of affordable and trustworthy outsourcing solutions for household tasks remains an important strategy to cope with competing resource claims from, and provide support networks for parents. Domestic outsourcing (van der Lippe et al., 2013; Epp & Valagaleti 2014), is an important way of coping with conflicting time demands, and manifests through ready-made meals, baby-sitting, household cleaning and maintenance. The availability of low-cost substitutes for household work may enable higher parental participation in the labour market (Cortes & Pan, 2019).

### Policy and societal implications

An awareness of how families respond to resource scarcity can improve the effectiveness of policies. Our research suggests that policy responses should take into consideration the dimensionality of important life events, the type of resource scarcity (time, money, space) being experienced and levels of chronic resource scarcity. Importantly, the effects of time and space scarcity should be attributed the same prominence as material deprivation due to money constraints.

Childbirth stands out as a life event that triggered both multiple forms of resource scarcity (time, money, space) and other transitions (e.g. changes in employment status, house moves). These experiences are instructive for both government policy makers and employers. Beyond one-off statutory maternity and paternity leave, families are likely to benefit from a wider range of policies designed to alleviate resource scarcity triggered by childbirth. Corporate child care programmes may assist parents in identifying affordable options and even providing financial assistance. Government funding can be provided to schools and local communities to provide child care in convenient locations to bridge space and time constraints. Other life events, such as chronic illnesses may be addressed more effectively by employers and policy makers by considering their resource implications on the patient and their families.

Time scarcity has significant consequences for families. Time poverty is known to have severe and wide-ranging impact on well-being, physical health and productivity of individuals (Giurge et al., 2020). Our study demonstrates that many families are time poor. Government service providers and businesses can improve the welfare of entire families by identifying and minimising administrative and idle times (e.g., involuntary periods between tasks, meetings, assignments) for service recipients and employees that do not further organisational objectives. Employers may partner with a variety of service providers to facilitate household tasks for their employees, such as grocery delivery, laundry and car services, and they could even provide consulting services to help employees with planning for significant life events. The use of technology can enable resource substitutions via the gig economy. Employment opportunities (e.g. Uber, Lyft) or social enterprises may provide flexibility in work hours, reducing conflict with child care responsibilities, or enable families to earn money by sharing space in exchange for money (e.g. Airbnb).

We also note the importance of chronic resource scarcity when considering policy and societal implications. Research suggests that childhood resource scarcity can shape adult responses (e.g., Griskevicius et al., 2011; Thompson et al., 2020). Our findings suggest that extra support is needed by families experiencing severe chronic resource scarcity as they balance child care with work, and cannot access paid support at the same level as mild chronic resource families. Policies that facilitate provision and maintenance of safe housing will not only alleviate space scarcity, but may have a range of positive effects on families. Indeed, families report multiple health and safety issues due to leaks, damp and mould across apartments, driven by lack of property maintenance (Hewitt, 2021).

At a broader level, our study offers valuable knowledge into how families as important constituents of society (see Haenlein et al., 2022) respond to resource scarcity - a substantial life issue. By studying resource scarcity from a family perspective, this paper also challenges the boundaries of traditional marketing research (MacInnis et al., 2020).

## Directions for future research

In this research, we examine how families respond to resource scarcity and interview parents from multiple family types, representative of the national population on numerous criteria (two-parent and single parent families, ethnicity, annual income, parent age). Our informants also encompass contemporary family types such as single dad, same-sex partners, blended and mixed-race families. Consistent with prior studies on family consumption and decision-making (e.g. Epp & Price 2011; Epp et al., 2014; Epp & Velagaleti, 2014; Thomas & Epp, 2019), our understanding is confined by the nature of our research context. We provide a first understanding of family responses to resource scarcity in a developed economy, where intensive parenting is prevalent (Gauthier et al., 2020). Intensive parenting is emotionally demanding, time consuming and child-centred. It is an approach that encourages substantial time, money and space investment to exercise appropriate parenting (Hays, 1996).

We recognise that other family types may have differing needs, resources and not embrace the same decision calculus. Further studies are required on families from heterogeneous populations such as the homeless (e.g. Cherrier & Hill 2018), vulnerable consumers (e.g. Hill & Sharma 2020), teenagers (e.g. Banister et al., 2016), or those from subsistence economies (e.g. Upadhyaya et al., 2013). Additional research is also needed in cultures where extended and/or patriarchal families are prevalent (e.g. Lien et al., 2018; Edirisingha et al., 2022), or where life events are handled differently (e.g. Western versus Eastern cultures).

For example, families in subsistence economies earn low incomes, lack access to basics such as food, water, education, healthcare, transportation, and have limited consumption options (e.g. Viswanathan et al., 2021). At the same time, they may also draw upon densely networked communities to offset resource scarcity (Viswanathan et al., 2008). In some cases, children may play a significant role in household tasks (e.g., baby-sitting, fetching water – see Hunter 2006). In other cases, females lead the improvement of the family’s resources (e.g. Nawrotzki et al., 2014), or spend more time doing household labour for their family’s wellbeing at the expense of paid employment (Giurge et al., 2020). Families may take the role of subsistence consumer merchants and run stores, sell home to home or in open markets as a means of generating livelihoods (e.g. Upadhyaya et al., 2013). Governmental, non-governmental organisations and social services may partake in helping families navigate scarce resources (e.g. Upadhyaya et al., 2021). As an initial attempt, our research thus describes mechanisms and provides knowledge for future research to recognise differences in other contexts.

Similar to Epp and Velagaleti (2014), Thomas and Epp (2019), we consider parents as the primary resource generators and allocators in families, as informants given the sensitive nature of our inquiry. We acknowledge that excluding children, especially older ones, may under-report goal conflict and misrepresent negotiation processes (e.g. Kerrane et al., 2012). What parents see as the “collective good” may not be perceived as such by children, who are at a crucial stage of development (e.g. John, 1999). Therefore, future research could look into differences in responses by sampling families with older children such as teenagers only. Extension of this research in subsistence economies may require that children are involved, given their likely greater engagement in the daily running of their households, through labour or house chores, potentially at the expense of full-time education. Such involvement may be facilitated by alternative methods such as participant observation or ethnography.

Consistent with MacInnis et al. (2020), there is a need to go beyond implicit boundaries guiding current marketing research and further investigate the effects of religious, ethnic, racial, minority affiliations and the elderly on responses to resource scarcity. Dietary requirements, specific rituals based on ethnicity (Rossiter & Chan, 1998) and religion (Cleveland et al., 2013) may impose added complexities in responding to resource scarcity. Crockett (2017), for instance, researches the consumption strategies of black, middle class consumers in the US and notes differences with the majority population. As the elderly navigate consumption to illustrate their identity and agency (Barnhart & Penaloza, 2013), research needs to investigate how consumers at this stage of the life cycle respond to resource scarcity. In addition, given that childhood SES is more predictive than current SES (Griskevicius et al., 2011; Thompson et al., 2020), researching the effect of childhood SES on family responses to resource scarcity may enhance our theorisation.

While we were able to discern differences in families’ short and long-term responses to resource scarcity, it is more difficult to isolate the effects of other dimensions of life events. Families may respond differently to life events that are positive versus negative, voluntary versus involuntary, happen one-off versus frequently (see Brammer, 1992). Researching the nature and experience of family responses as they work through other dimensions of life events is beyond the scope of this paper. It is especially challenging to isolate the various aspects of life events because families often encounter life events concurrently and consecutively. Given the nature of life course, such investigation is likely to require disentangling via experiments. Finally, we do not examine the emotional repercussions or stress, of continuously responding to resource scarcity. According to Mathur et al. (2008), life events are stressful and influence consumption. Therefore, we suggest the need to study the effects of responding to resource scarcity on family stress and emotional well-being.

## Conclusion

Our paper fills an important theoretical gap and investigates how families respond to resource scarcity. We illustrate that multi-dimensional, concurrent and consecutive life events trigger situational resource scarcity by creating a mismatch between resource availability and demands. Our research highlights adjustments in short-term consumption and long-term resource investment. Responses occur within the context of family interactions, presence of support networks and families’ chronic resource scarcity. In closing, we hope our paper serves as a springboard for research on the substantial topic of family responses to resource scarcity.

### Supplementary Information

Below is the link to the electronic supplementary material.

ESM 1 (DOCX 481 KB)
