# Supplementary material for: Family responses to resource scarcity
Source: J Acad Mark Sci. 2022 Jun 30:1–31. Online ahead of print. doi: 10.1007/s11747-022-00882-7 (PMC9245874; doi:10.1007/s11747-022-00882-7)
Supplement: Supplementary file 1 — (DOCX 481 KB) [file 11747_2022_882_MOESM1_ESM.docx]

Web Appendix 1: Steps Adopted in Thematic Analysis

| **Steps** | **Process Applied** |
| --- | --- |
| i. Preparation for fieldwork | - Review of family decision making, consumption and resource scarcity literatures - Creation of semi-structured interview guide based on family decision making, consumption and resource scarcity literatures |
| ii. Familiarizing with data | - All interviews transcribed verbatim - Transcripts reviewed to identify initial observations - Listen back to full or, relevant parts of recordings to clarify understanding of transcriptions |
| iii. Creating initial codes | - Initial / first order codes identified based on interview guides, transcripts and recordings - Coding template developed. - Interview data coded using developed template - First order codes triangulated by family and across resource type |
| iv. Identifying themes | - First order codes analysed to seek over-arching, second order themes. - List of first order codes organised into second order themes - Codes and themes checked for consistency - Create and triangulate second order themes by family and across resource type |
| v. Reviewing themes | - Compare first order codes and second order themes - Merge second order themes into aggregate themes |
| vi. Defining and naming themes | - Refine linkages between first order, second order themes and aggregate themes - Create Figure 1 (Appendix 1) as final coding structure |
| vii. Writing up analysis | - Create meaningful representations of thematic analysis through a conceptual framework (see Figure 1) - Further checking, editing and refinement of concepts |

Source: Adapted from Braun and Clarke (2006) and Raja et al. (2018)

Web Appendix 2: Responses to Resource Scarcity (Consumption and Resource Adjustments based on Family Chronic Resource Scarcity)

| **Family Characteristics** | **Responses to Resource Scarcity** | | |
| --- | --- | --- | --- |
| **Family Type: Single Parent** | **Time** | **Money** | **Space** |
|  | **Adjust total consumption** | | |
|  | Jackie (MOD): I've had two weeks this year where I've had to do evening webinars, [with] the kids around [and not with their divorced dad] … actually, I've had to rely on [devices] because I need to be focused for two or three hours … | Brenda (SVR): …We had a bit of a bad time … he [divorced husband] wasn't paying any money at first … that was probably the worst time … I'm not one to ask my family … [for money] ... [I would] cut down on shopping and buy [less] … I would find cheaper stuff, look around … not spend too much … | Candice (SVR): I [had] been with obviously, my husband for 18 years … and the kids knew nothing different than being with my husband, and me … and going from living with mum and dad to live with just mum at Nan's house was the hardest for them … |
|  | **Adjust consumption across family members** | | |
|  | Ana (SVR): Sometimes, instead of going shopping, I just lie down [because of my heart condition] ... that makes things delayed ... it's chaos, because for me, there's always food in the fridge, but [my son] is specific ... he wants to eat certain things ... and I have to rush shopping …. when you have two parents, then obviously, they support each other ... | Candice (SVR): … Kids constantly need new clothes, because obviously they're getting bigger … so I sacrifice my clothing for my children … I've got clothes that I'm wearing that I wore five years ago, because … I would rather my kids have new clothes, and shoes and things … if they need a haircut, I love [prefer] the £10 to go on their haircut, than £20 on mine … so I neglect my hair … I sacrifice a lot … | Monique (SVR): I [and kids] try to not have too much [belongings] ... if it's too many things, I am always giving to charity … [to make space] |
|  | **Substitute resources invested** |  |  |
|  | Brenda (SVR): I work part time around the hours of school … | Candice (SVR): At the moment, I'm [on] Universal Credit [government support] … obviously when [1-year old] goes to school, then I go back to work … I'm hoping I can get a job in the school because I'm qualified … [and be home on time to look after my 4 children] … | Candice (SVR): … We were not expecting to be with my mum for 15 months … I thought my [divorced] husband will call us back … it's a long time to be stuck in the smallest room in the house with three children … I could not afford a place of my own with my part-time income |
|  | **Adjust resource investment across family members** | | |
|  | Ellia (MOD): Because I'm also busy with my work [as a single mum] … I'm not there 24/7 to support him [youngest son] ... But the eldest one [son] is able to do it … [he] has been helping me a lot when it comes to supervising [the younger one], even [at bed time]. | Lily (SVR): [I said to my son] … until mum gets her whole salary, we're gonna have to make sacrifices … my son understood … he now has a part-time job … | Monique (SVR): We've got a two-bedroom flat ... my daughter got [her] room, my son got [his] room and I sleep [and work] in the living room… When [work is busy] my living room looks like a warehouse ... I want to do it very quick ... I ask my kids for help ... [my son] cannot, but with [my daughter], I pay her some money... and she’s helping me … as a single mum, I have to do everything myself … |
| **Family Type: Two-Parent** | **Time** | **Money** | **Space** |
|  | **Adjust total consumption** | | |
|  | Angelina (MILD): I didn't have time to finish books off ... because when you've got kids, your time is very fractured ... once or maybe twice a year, I would go away [in a hotel] for sort of three to five days, to just spend a few solid days working on the draft of a manuscript … [Max or grandparents will handle the kids] … | Jake (MILD): As a family, … we have tried to really rationalise our expenditure … [as mum stopped working] … | Stephan (MOD): We've [Esther and I] moved stuff outside into the garage ... we've had external storage … |
|  | **Adjust consumption across family members** | | |
|  | Jack (MILD): When I was finishing my Masters, … [I had a lot of] work to do on my dissertation, Mark [other parent] was busy. I took him [son] to soft play like 10 am to 5pm, [he was] … having food there … and he played … | Ritika (MOD): They're [kids are] very easy going. Kids will ask 'Can we have that?' [I will say] … the price looks expensive … on Amazon [it's] cheaper. 'That's fine Mommy' … | Vicky (MILD): Robin's parents came to stay, and we put them up to stay in a hotel, you know … we did have a sofa bed … but that's not really welcoming … |
|  | **Substitute resources invested** | | |
|  | Eloise (MOD): Whereas before I work from home, I'm always available. But now that I've returned to work [in a nursery] ... it does make a difference, especially if your partner has to be outside of the home as well .... So, ... [eldest son] watches [over youngest], because he is at an age where he can ... | Anand (MILD): The original plan was that she [daughter] would start nursery … we were lucky that my mom and dad agreed to come and stay with us ... | Rose (MILD): [years after our move] … we converted our garage, … that's now my study ... we've got gym equipment, Charlie's got a dartboard up, a table tennis for the garden … |
|  | **Adjust resource investment across family members** | | |
|  | Vicky (MILD): Luckily, we had my mum, [who] could help out ... because Robin was working in London ... [when] I would be rehearsing on weekends [for my theatre production], [Robin] would definitely have the children. … | Angelina (MILD): … Max had gone down to a four-day week, and his work was less certain than it was. I've started to run online writing workshops to make a little bit of money ... | Alan (MOD): We're in somebody else's house [over 8 years now] …we share the kitchen, bathroom ... we're constantly dancing around to try and not be under anybody else's feet … we go to the bathroom first, get washed, dressed before [Beth's parents] start to do their bits … the same with cooking … We are still saving for a mortgage |
| **No of Income Earners: One** | **Time** | **Money** | **Space** |
|  | **Adjust total consumption** | | |
|  | Ritika (MOD): … the collection point is closed on Saturdays and Sundays … [the only time] I can collect is between Monday to Friday … [my parcel has] been there for two weeks now and I don't get chance to go because of the time table for the kids [11plus] tuition homework … my husband is constantly working, so he can’t help me either … | Priya (MOD): We want to buy house but … [with] one person earning it's a bit hard for us …so we are saving and renting for now… | Ash (SVR): Children need a big room … we don't have car … [but can't move out because] everything is local… [to dad’s job] |
|  | **Adjust consumption across family members** | | |
|  | Shruti (SVR): [Due to] time implications of looking after child with allergy, me and the kids usually eat leftovers from night ... for lunch … our son’s food needs to be prepared in a specific way and we cannot rely on take-away or restaurant food … my husband helps with cooking and looking after children when he comes back from work … | Tom (MOD): We budget for our family holidays … look at it, and then decide, … we can't go there because that's ridiculous money per night with one person’s salary… Let's go to this other place …. | Ash (SVR): [Doctor ask to] change [water softening equipment to help with child skin condition] ... we say that this is not our own house … children need a big room …we don't have car … [but can't move out because] everything is local … [to dad’s job and mum does not work] … |
|  | **Substitute resources invested** | | |
|  | Albert (MOD): I recall a moment when really, really the time was tight. I was working two jobs. I was driving the [bus] and I was managing a restaurant in the evening ... And ... we barely saw each other [wife, Faith, a full time stay at home mum at the time] ... | Daniel (MILD): If she [mum] were to go to work, we would be literally paying for out of school club, nursery, so it didn't, really make sense ... let's wait for the kids to start school … | Ian (MOD): … We would be already probably in some lovely one-bedroom house, … [that’s] everything we can afford [with my income] … but you sacrifice … unfortunately, house prices are very high here … so it's kind of pointless … the boys would want their outside space to play …. [the one-bedroom does not work for us] … |
|  | **Adjust resource investment across family members** | | |
|  | Vicky (MILD): [Time] … made me think about ever doing it [the production as a hobby] … And I can’t say I have a job … you can be practising and learning your lines in the day when they're at school ... [but for] Robin, certainly when I was doing that play, it was like, Oh, that's another afternoon gone, where you would be doing work, rather than childcare | Faith (MOD): Because of me not working ... to get mortgage was going to be tight  Albert: Selling our old house was the [only] way that I'm going to buy the [new] house ...and I need to have a little bit spare for emergency, and to fix the [quite old] house ... | Vicky (MILD): If Robin had to work at home in our old house ... [it] would have been really tricky, ... but crafting is something I'd like to do maybe as an income source one day ... so that means, having a lot of supplies, and having the space to do it, ... |
| **No of Income Earners: More than One** | **Time** | **Money** | **Space** |
|  | **Adjust total consumption** | | |
|  | Alan (MOD): We both work … we get lost in our day ... we set reminders in Alexa to do things …. technology enhances, … and supports [our lives] ... | Stephan (MOD): I don't really want to start painting at seven o'clock at night [after work], and [there is either] a birthday party, or a football match, or something [else] on [at the week end] … [everything] depends on both our work commitments, … [The] financial element ... made quite a big difference. It's worrying about ... how much it was going to cost, [whether] we can do it, and ... [if there is] enough food to eat every night of the week … | Rose (MILD): ... We left the house [during my maternity leave and the summer [school holidays] and they [builders] could ...do everything. ... without spending on renting another property ... moved out to my parents’ house .... Charlie could commute into his work … |
|  | **Adjust consumption across family members** | | |
|  | Summer (MOD): We usually take our [work] holidays around the school holidays ... we alternate [to cover child care] … | Navin (MOD): Sometimes, kids ask [to go] somewhere on holidays …. [if] it's not enough money …. we'll say maybe next year | Alan (MOD): …We've got crates stacked up in rooms … a shed, in the garden full of our stuff … [even if we both work] we haven't been able to bring [daughter] up in the environment we wanted ... being able to spread her toys out ... they are stacked up in crates |
|  | **Substitute resources invested** | | |
|  | Kate (SVR): When they [kids] were much younger, I wasn't working. It was much easier. Because even though financially we struggled, but I was always there …Now that I work, there is a lot more juggling | Eva (MOD): My sister used to live with us before … so she looked after [my autistic son] when I was working [and I didn’t have to pay for child care] | Albert (MOD): I really wanted to actually keep the [old] property and buy this [new house] as well, [as] I was being asked to rent the [old] property from British Airways ... it was a good deal ... [but] I couldn't afford it .... |
|  | **Adjust resource investment across family members** | | |
|  | Julia (MOD): … We kind of fill the gap for each other …. we both are working, we have to coordinate  David: [Its’] a bit different because my job is very intensive, it could be weekends, it could be Christmas Day …. It could be nothing for two months. | Eloise (MOD): We pay the big portion of the month's bill out of my salary [as I get paid monthly] ... Matt gets paid weekly ... I do like a big monthly shop … we need food shopping topping up [weekly from Matt's wages] … We share responsibility in a different way | Eloise (MOD) I needed [elder son’s] bedroom, to put a child to sleep ... [during school holidays, when partner was at work], I'd have to say to him, [if] he could go out of his room for an hour ... so, he knew his mommy's job [childminder]... |
| **Socio Economic Status (SES): Low** | **Time** | **Money** | **Space** |
|  | **Adjust total consumption** | | |
|  | Lily (SVR): Sometimes … I would come home like 12 30 at night, and they [child and ex-partner] will look at me and say, “what was for dinner?” Can you believe it? I just arrived [after a full shift] … we've got a Marks and Spencer, which I don't like buying because it's so expensive … but I will buy something on “reduced” [cheaper price due to expiry date] and cook … | Kate (SVR): I suppose we don't really go on family holidays because we generally cannot afford them … | Ash (SVR): We don't have car … but [at the] end of the day, we keep everything local … |
|  | **Adjust consumption across family members** | | |
|  | Ellia (MOD): I've got my meetings ... And then [its] late ... I have to rush to pick him [son] up, and say ... Today, we just gonna have something [easy] because I didn't have time to cook ...  Son: Mommy, it's fine ... let's have tacos ... | Marla (SVR): We don't really go anywhere ... I just buy basic presents for the kids [and] my husband … We don't really spend money now … the rent is so expensive ... more than half of the salary … | Ana (SVR): … No space for my son to exercise …he asked me to buy a stationary bicycle … we have nowhere to put it … We struggle with space … I had to move the computer, dining table, store all my documents online … |
|  | **Substitute resources invested** | | |
|  | Lily (SVR): My dad used to help me a little bit [with child care, when I went to work] …. because the family was supportive … [otherwise] …. I just had to close the door. I had nobody to look after [child] … she had to be responsible since she was like 13 or 14 … | Candice (SVR): [I was] stuck in the smallest room in my [parents’] house with three children … [for free] being pregnant … I didn’t have a place of my own [after my divorce] … | Kate (SVR): I would have liked to have a cabin in the garden ... for me to do my homework. And just have that bit of space and time when I need to do lessons or ... time to do essays ... but too many things financially come up. And I didn't really get that opportunity … I have been struggling … |
|  | **Adjust resource investment across family members** | | |
|  | Monique (SVR): I do everything by myself ... my daughter [12 years] helps me with cleaning toilets, kitchen or dishes, ... with cooking, they help me sometimes, because I cook every day … their [divorced] dad comes to visit only … | Gabriel (SVR): So, our eldest son, is working ... we are getting him to start paying some rent money now ... he does part time work part time college... | Candice (SVR): [After my divorce] … my three kids [and me] were in one … small, tiny, little room … I had to have a bunk bed with a double on the bottom … me [pregnant] and my daughter [at] the bottom, and my two boys actually shared the top bed …. |
| **Socio Economic Status (SES): High** | **Time** | **Money** | **Space** |
|  | **Adjust total consumption** | | |
|  | Rose (MILD): We spend more, ... the nursery ...is probably more expensive [due to] longer hours, we get take away, because we don't have time to cook ... But, you choose to work ... you also earn more to compensate. | Daniel (MILD): …We wanted to travel every half term ... but because money was a problem, then we would pick one or two half terms… | Navin (MOD): We got three bedrooms … one of the bedrooms is [the] office … most of my time [is spent] in this one bedroom … Boys want to play football, basketball inside… most definitely they can't …[and] outside there is not [much] space to play … |
|  | **Adjust consumption across family members** | | |
|  | Louise (MILD): We've got two [kids] birthday parties on Saturday … and my sister's graduation party … to fit that in, to make everyone happy is an absolute nightmare … Jamie's going to run to the parties … and I set up with the kids for my sister's party, and we meet up later on … it's that juggling act | Stephan (MOD): [I do not] want to go on holiday and incur debt … we might go for days out to the local theme parks … or actually, we would just end up doing things around the house …. | Angelina (MILD): We had an arrangement before where ... I had a nice big study to write in. Then Max's office shut so he's working from home permanently.... he needed a place to work that was kind of quiet and away from the kids … so he took over my study ... |
|  | **Substitute resources invested** | | |
|  | Louise (MILD): We had an au pair to look after the kids [and the house] as we both worked …. | Vicky (MILD): We have to decide whether we wanted [private education] for our children .... money would be a factor ... on top ... it would have been, a lifestyle change … If you go to private school, there's lots of additional costs …. then I started to look down the grammar school routes ... [free school, admission based on exam score] … | Rose (MILD): We were living in a flat, …. which was fine, but we always wanted to be in a house ... we made a decision to move ...to have more space …. we converted our garage, … that's now my study ... we've got gym equipment, Charlie's got a dartboard up, a table tennis for the garden … |
|  | **Adjust resource investment across family members** | | |
|  | Louise (MILD): We had an au pair to look after the kids [and the house] as we both worked ….  Jamie: When the au pair left, you reduced your work hours … and I’ll spend more time at home … | Summer (MOD): I work Monday to Thursday. When I was looking for nursery for [youngest], they could [do] only four days … I work when all the [four] kids are at [nursery, primary and secondary] school …” | Reema (MILD): … before [the] baby, the nursery was the study … we had to convert one of the other rooms into a study …. |
| **No of Children: One** | **Time** | **Money** | **Space** |
|  | **Adjust total consumption** | | |
|  | Amelia (MILD): I struggled with trying to cook three meals a day for three of us while entertaining ... a small baby… I decided that it was acceptable to sometimes use a pouch of pre-prepared food … | Brenda (SVR): I'm not one to ask my family … [for money] ... [I would] cut down on shopping and buy more things that are better for you … I would find cheaper stuff, look around … not spend too much … | Lily (SVR): … Sometimes you have to go to the car to make a phone because our [small] home was noisy …. |
|  | **Adjust consumption across family members** | | |
|  | Brenda (SVR): I can do a lot more shopping and things [when daughter goes to see her dad] ... It's quite hard to take [her] shopping … | Alan (MOD): It would not be a sensible thing to spend £2000 - £3000 on a family holiday for the three of us for two weeks in the sun … | Lily (SVR): When my only child decided to come and live with me and my ex-partner … he had to sleep on the sofa bed for a while until we moved…. |
|  | **Substitute resources invested** | | |
|  | Vera (MOD): … [Before giving birth] I used to be a volunteer, deputy editor [for a magazine] … I had to give that up [at child birth] … its’ gonna be a while … she needs to grow a bit … when she starts nursery, I'll go in and hopefully maybe even work again a bit | Jake (MILD): The sheer cost of [childcare makes you] question [its] utility ... the potential possibility of having to pay someone, a significant amount of money, when you don't think they'll do a better job than you're ... Amelia, decided to give up work … | Reema (MILD): Before [the] baby, the nursery was the study … we had to convert one of the other rooms into a study …. |
|  | **Adjust resource investment across family members** | | |
|  | Reema (MILD): The original plan was that she [baby] would start nursery … I was ill after [she was] born ... my mother in law did all the cooking ... [Anand, husband] did the cleaning, shopping, washing up, or whatever it was … | Brenda (SVR): I work part time around the hours of school … her [divorced] dad and my mum also look after her when I can’t … | Jake (MILD): Before [the baby], we were very well stocked in a house … then she turned up … [we had nowhere] to keep all the gifts, equipment, prams …  Amelia: [After the house move] … he has a study … we have a dining room |
| **No of Children: More than one** | **Time** | **Money** | **Space** |
|  | **Adjust total consumption** | | |
|  | Jackie (MOD): I've had two weeks this year where I've had to do evening webinars, [with] the kids around and actually, I've had to rely on [devices] because I need to be focused for two or three hours … | Marla (SVR): [If the kids want games] … the brand new one is £30 pounds and a second hand one, £7, I buy the £7 one … [younger son] got a lot of toys from [elder] when he was the same age … [that] he can play now | Kate (SVR): … only me and my husband can’t have a room on our own ... if you want to watch the news, they [children] don't like it ... and we don't have a TV in our bedroom and neither do the girls … the family shares … |
|  | **Adjust consumption across family members** | | |
|  | Julia (MOD): As a family, we are kind of restricted by the respite [support to look after disabled child] we get … it's kind of difficult to bring [son] out because he's quite big now and … is a challenge … for us to be able to go out as a family, usually, we put him for a weekend or something like that… [with a respite family]. | Ritika (MOD): They're [kids are] very easy going. Kids will ask 'Can we have that?' [I will say] … the price looks expensive … on Amazon [it's] cheaper … they will say: 'That's fine Mummy' … | Ana (SVR): … he [son] asked me to buy a stationary bicycle [for exercise] … we have nowhere to put it, we struggle with space, I had to move the computer, dining table, store all my documents online … |
|  | **Substitute resources invested** | | |
|  | Julia (MOD): [Looking after second child was] easier … I had a long maternity … And he [David] didn't work much … he stopped work for two years … So, I look after her for the first one and a half years … and then he took over for two years, and then at three and a half, she went to nursery. | Albert (MOD): We had one person working, two young kids ... we had to cancel the holidays [abroad] that we wanted .... we had to sacrifice ... It was worth it to be in greater place ... | Eloise (MOD): I [had] made plenty of space for the [minded] children's stuff, but as my children [two boys] got older, they didn't need those things [toys] ... you sort of think I haven't got a big house ... I'd probably still be childminding if I had a [bigger space] … I had to swap jobs |
|  | **Adjust resource investment across family members** | | |
|  | Kate (SVR): Whereas I wasn't at work today, I was at home with them [4 kids] ... tomorrow I will be at work ... but my husband will only be able to have half his usual sleep ... we feel guilty if my husband's asleep, they [kids] get bored without having a parent around. And we do leave activities and everything out ...they're [kids] only on their own for about three and a half hours … | Albert (MOD): … [we] concentrate on accommodating kids ... and then look for ourselves … we don't hold back ... I am a good saver ... not obsessed with expensive stuff or going out to dinners every weekend or night out ... but we want [our children to go] to university and we are saving for it … | Anand (MILD): When we were buying our car, we had to buy, a very big practical car… [to accommodate 2 babies, car seats, buggies, … |

Notes: Severe Chronic Resource Scarcity: SVR; Moderate Chronic Resource Scarcity: MOD; Mild Chronic Resource Scarcity: MILD

Web Appendix 3: Responses to Resource Scarcity based on Time Factor

| **Time Factor** | **Responses to Resource Scarcity** | | |
| --- | --- | --- | --- |
|  | **Time** | **Money** | **Space** |
| **Decisions about Consumption: Short Term** | Julia: It's kind of difficult to bring [8-year old disabled and autistic son] out because he's quite big now and is a challenge to be out of house … for us to go out as a family, [we rely on a respite / support family] … the only time [is when] kids go to bed and all the work is done … we might watch a film [at home, on Netflix, rather than going to the cinema] …  Mark: We might plan a Saturday day trip, and then I can't go [due to last minute work commitments] … so I stay at home working … Jack and [son] go to town …  Jack: [son] loves soap from Lush [cosmetics shop] … you spend £10 and try to compensate … | Ana: Mary needed new trainers because she grew out of [old ones] …[her] dream trainers [were] £60 in the shop … I found them on Vinted [web site for used items] for £6 or £7 ….  Ellia: I only had £20 and I had to pay the school meals, ... I had to call the school, to lend me some money … | Daniel: We used to live in a house that was much, much smaller, [than] a two-bedroom house … [Due to lack of space], on Saturday [we] would go to soft play ... then on Sunday, we could take them shopping, [and] even buy some presents for them …  Tom: [The house, which I bought as a bachelor, is very small for the three of us] … we can't hold events here … we were going to have a barbecue [for one of our birthday’s] in the garden … but we moved it to a restaurant … |
| **Decisions about Resources: Long Term** | Daniel: If she [Ellie, mum] were to go to work, we would be literally paying for out of school club, nursery, so it didn't, really make sense … let's wait for [both] the kids to start school …  Faith: For so many years, I didn't work … [Albert] had to be the main [income earner, as I had to look after the kids] ...  Albert: Now, Faith works between the hours of school ...  Faith: [If I didn't sacrifice, I wouldn't] have two years of experience in accounting, [but rather] 12 ... | Reema: We don't have the money right now [to improve the whole house] ... we'll do it in five, six years … when we've been able to save up enough ....  Jake: Amelia, deciding to give up work [due to the baby, was a] … household altering situation where you're probably reducing your house joint household income by 50% or 40% … [but] we got a bigger house [by moving] to a much cheaper area ... and maintain the setup that we were already in [same income to costs ratio] ... | Paul: Four and a half years ago I moved in this flat … [as] I [previously] had only one room, bathroom, kitchen, a hallway and a bit of garden … [I applied to the council] and they gave me a bigger one [flat] …  Alex: [Space is] becoming an issue … we finished the refurbishment a couple of years ago … and it's [already] getting to the point that [we need] a bigger house … [we want] to make a considerable jump … we don't want to make a small step, which is not worth the time and effort … we want something considerably bigger, [for which we have to wait] …. |

Web Appendix 4: Responses to Resource Scarcity based on Family Interactions

| **Examples of Family Goals/Priorities** | **Responses based on Elements of Family Interactions** |
| --- | --- |
| **Education**  Navin: We want to put more time towards the children education, irrespective [of] buying a house …  Vicky: Money is definitely important in everything we do … education also is … | **Family Flexibility:**  Navin: Priya doesn't work ... [if she] has to work [she] will divide her time between office, kids and household ... if we get money, [we] can buy a house [but] ... means, less time for the kids’ education ….  **Negotiations, tensions & challenges:**  Navin: When the kids were younger, we used to have a little bit more time …. Now they are used to the gadgets and class [levels] are increasing … [we] need to put more effort to make them learn, understand, ….  Researcher: Who has more control over the kids’ education?  Navin: It is a matter of mutual agreement between two of us [parents] …  Researcher: How do the kids feel about that?  Priya: They sometimes hate, sometimes love, sometimes cry, … we sometimes bribe them, sometimes get angry ... it's a mixture of things ...  Vicky: We have to decide whether we want [private education] for our children .... money would be a factor ... on top ... it would have been, a lifestyle change … If you go to private school, there's lots of additional costs [in addition to the semester fees] … then I started to look down the grammar school routes ... [free school, admission based on score from competitive exam] ... [I have been] looking at the location of some of these [grammar] schools as well …. there is a differentiation in the fees … it can [costs up to] £1000 pounds to sort of travel there  **Domains of Control:**  Priya: … Only I'm going to do housework … and he will do job … [in weekends, we] … mostly will spend time with the kids and shopping ... sometimes we'll play together ….  Navin: ... If I'm free, I take care of kids' teaching ... and she [mum] mainly concentrates on the cooking ... |
| **Savings/Budget**  Marla: We [are] always saving …  Navin: Savings are important for us ….  Ellia: Budget and savings …  Ana: I'm economising a lot …  Monique: I will never spend money for [unneeded] things  Ellia: Budget and savings …  Kate: Time and budget management ….  Stephan: I do not want to incur debt … we will sit down and come to a compromise … | **Family Flexibility:**  Marla: [I started working] straightaway [after childbirth], because it's your own business … I work from home ... it's more flexible ... it's not like I go to a workplace 9 to 4 ... if I'm home, I do the housework, …  **Negotiations, tensions & Challenges:**  Navin: We want to buy house but … [with] one person earning it's a bit hard for us…  Priya: Sometimes kids ask [to go] somewhere on holidays …. [if] it's not enough money …. we'll say maybe next year … we try to save it up for the holiday … Because [once] kids decided they want to do certain things, you can't really change their minds… sometimes they [kids] will ask [for] small things [which] is okay … My eldest son had his birthday last month … we bought [what he wanted] ... but not more than that …  Ellia: I go [food shopping] on Monday morning, because Sunday we'll discuss and Monday morning I'll pick up things for the week ... Every Monday they [supermarket] put fresh food ... that's one of the reasons I go and sometimes in the evening. If I want to get something at a very good price, I'll go ... before they close ... what you get for £2.50, they do it for £1 ....  Ana: … [I'm the] main earner and I'm paying all the bills ... it will change at [son’s] birthday ... there will be no money [government help] coming in, because of his age … we have to be much more careful with the money … I think when he [son] needs [to earn] money … he's clever enough to take a decision [on] how much time he can spend earning money, and how much he needs to study …  Monique: I will never spend money for [unneeded] things … it's not necessary … [their dad] feels guilty after our divorce, … If I will tell him that [kids] need new shoes or something, or I need washing machine, he's gonna buy it … [my daughter] tried to [argue], who's got the new IPhone? Why her current device is not good ... its old fashioned, whats'app not gonna work ... she didn't see any reaction from me, she said that she can [start] saving ... she was thinking she's gonna manipulate him [her dad] and he's gonna pay for it … he didn't  **Domains of Control:**  Ellia: All of us we do, [input on the weekly meal planning] but I'm the one who's going to decide, for example, lasagna on Friday or Saturday, But, when it comes to the budget, of course, I'm the one who has to manage ...  Kate: Food shopping is generally a bit of a struggle ... and school dinner money, seems like a lot when you pay out … We tried [swapping school dinners for packed lunches] ... we didn't think it was cheaper ... When we buy the things, our kids feed on it, and then we have to go back to the shop ... so time and budget management, knowing there's something there ... like the school dinner, the safety net ... we have enough money to generally pay for all the basic, it's just, there's no real pleasures, or fun stuff ... As the mum, I think I've got to manage the kids more … and I know what works really ….  Stephan: depends on the necessity of the spend ... kids would come first ... in most instances, and that would mean that we might forego a luxury to provide them [kids] with a luxury ... and certainly, we would forego a luxury to provide them a necessity ... and, they might have to forego a luxury to provide us a necessity ... but when the scales are flat, they will always win. |
| **Health & Well-Being**  Julia: Having ‘me’ time and keeping healthy is very important for us …  Jackie: I think the kids are most important, but I make the time management decisions …  Summer: One of the things we don't compromise, is feeding … that is a priority … anything that benefits the health of the children …  Julia: Having ‘me’ time and keeping healthy is very important for us …  Rose: I'm always kind of looking after the kids. And I would come last in the list …. | **Family Flexibility:**  Julia: [We] plan and cook [healthy food] in the weekends for the week ahead … We have a big freezer and fridge … we are very organised … because we have no family here to help us … his parents are in Greece … my family is in Singapore … we used to have cleaners before the pandemic … I don't think they ever clean to our standard … But then it means that we take on everything ourselves again … the time is only 24 hours a day. It's just how you prioritise ….  **Negotiations, tensions & challenges:**  Jackie: The kids want to be on devices a lot of the time … it's a generational thing ... I always think time in front of a screen is wasted … I put screen time limits on them ... their dad [divorced] doesn't … that's conflict.  Summer: He [husband] may be at work and come home tired ... and I have to cook [and not get take-away]... I get the children involved, setting up table, tidying up, sitting room, doing their beds …we work as a team … He may cook for all of us to sit down, … enjoy … maybe I will tidy up or we all get together [and tidy] … We don't sit down and say, Oh, you have to do this or this person has to do that ... we work as a team …  Julia: People think it’s difficult being without holiday … and they suffer …. [for us] it's just something that we have to get on with …. like in school holidays, we cannot switch off … we have to be hands’ on for him [child] …. that's the reality …  David: If you have children, [you] dedicate to this … you surrender your life and schedule ….  Julia: [In] the same way they say that they haven't seen their family, but we haven't either …. we haven't been back to Singapore for 10 years … He hasn't been to Greece for 7 years … We [can] go to like four or five days but we can't go for longer … because there's no respite [charity/child support] longer than a week … even with the respite …we have to plan ahead and [still] it can get cancelled last minute and all the planning goes down the drain … The only things we can do is watch Netflix or stay local …  **Domains of Control:**  Rose: I would drive the kids in with me, and have them at nursery nearby, so I could stay at work later ... pick them up and drive them home with me .... |

Web Appendix 5: Resource Assistance from Support Network (extended family, friends, government, community services)

|  | **Name** | **Resource Utilisation** | **Resource Assistance from Support Network** | | |
| --- | --- | --- | --- | --- | --- |
|  |  |  | **Time** | **Money** | **Space** |
| 1 | Marla & Nick^**^ (SVR) | - Live in rented flat - Regularly spend time abroad during school holidays, and benefit from *extended family* support for child care | Regular | X | X |
| 2 | Priya & Navin^**^ (MOD) | - Live in rented flat - Benefit from occasional support from *local friends* if needed for child care; extended family live abroad | Occasional | X | X |
| 3 | Faith & Albert^**^ (MOD) | - Live in own house - Support from any *local*, *extended family*, and *friends* occasionally for child care; one-off occasions for space e.g. during home improvement; most of extended family abroad or geographically distant | Occasional | X | One-Off |
| 4 | Vicky & Robin (MILD) | - Live in own house - Occasional support from *local, extended family* for child care to support mum’s hobbies | Occasional | X | X |
| 5 | Ellia^*^ (MOD) | - Live in rented flat - No extended family within geographical proximity for child care; local clubs regularly help with dropping and picking children as mum does not drive - Extended family occasionally helps with financial matters | Regular | Occasional | X |
| 6 | Alan & Beth (MOD) | - Share house with *extended family* (mum’s parents) - No child care support from grand-parents due to old age and disability | X | X | Always |
| 7 | Angelina & Max (MILD) | - Live in own house - Occasional support from geographically distant*, extended family* to support mum’s writing retreats and substantial children monetary gifts | Occasional | Occasional | X |
| 8 | Kate & Gabriel (SVR) | - Live in own house - No support from *extended family* or *government* | X | X | X |
| 9 | Eloise & Matt (MOD) | - Live in own house - Some support from *local, extended family* for child care | Occasional | X | X |
| 10 | Summer & Harry^**^ (MOD) | - Live in own house - Some support from *local friends and* geographically distant*, extended family* when they visit | Occasional | X | X |
| 11 | Jackie (MOD) | - Live in own, recently downsized house due to divorce - Some support from *local friends,* geographically distant*, extended family* for child care | Occasional | X | X |
| 12 | Miranda & Alex^*^ (MOD) | - Live in own house; rent outside storage for work due to limited space in house - Limited support from geographically distant*, extended family* for child care | Occasional | X | Always |
| 13 | Shruti & Ash^**^ (SVR) | - Live in rented house; no support from *extended family* as they live abroad - High support from *community and local church* for day to day needs, including housing | X | Always | Always |
| 14 | Stephan & Esther (MOD) | - Live in own house - No support from *extended family* for child care | X | X | X |
| 15 | Brenda (SVR) | - Live in house, provided by the *government* - Regular support from *local, extended family* and *separated dad* for child care - Some monetary support from *dad* for child expenses | Regular | Regular | Always |
| 16 | Ana^*^ (SVR) | - Live in house, provided by the *government* - Some support from *divorced dad* for child care - Eldest son on minimum *government support* | Occasional | Occasional | Always |
| 17 | Anand & Reema (MILD) | - Live in own house - Regular support from geographically distant, *extended family* for child care | Regular | X | X |
| 18 | Monique^*^ (SVR) | - Lives in rented house - Monetary support from *dad* for child expenses - Limited support from *local friends* for child care | Occasional | Always | X |
| 19 | Amelia & Jake (MILD) | - Live in own house - Regular support from *local, extended family* for child care | Regular | X | X |
| 20 | Rose & Charlie (MILD) | - Live in own house; one-off support from family to share house due to home improvement - Outsource child care, household chores, meals - Regular help from *local, extended family* for housework | Always | X | One-Off |
| 21 | Ellie &  Daniel^**^ (MILD) | - Live in own house - Regular use of paid for, outdoor play areas to cope with limited space - No support from *geographically distant, extended family* for child care | X | X | Regular |
| 22 | Ritika & Rishi^*^ (MOD) | - Live in own house - Regular support from *local, extended family, friends and neighbours* for child care | Regular | X | X |
| 23 | Candice (SVR) | - Live in house, provided by the *government* - Regular support from *local, extended* family *to* look after younger ones; Complete reliance on *government* funds for *other expenses*; limited support from separated *dad*. | Regular | Always | Always |
| 24 | Louise & Jamie (MILD) | - Live in own house - Regularly rely on au pair for child care | Regular | X | X |
| 25 | Lily^*^ (SVR) | - Live in rented house - One off support for financial management from *church* - Regular support from *landlord* to reduce rent and avoid space constraints | X | One-Off | Regular |
| 26 | Paul^*^ (MOD) | - Live in house financially supported by the *government* - Regular support from *separated mum* for child care | Regular | X | Always |
| 27 | Mark & Jack^*^ (MILD) | - Live in own house - One off support from *geographically distant, extended family* or *local friends and neighbours* for child care | One-Off | X | X |
| 28 | Julia & David^**^ (MOD) | - Live in own house - Regular support from *charities* to look after disabled child for parents’ time off; regular use of *after school clubs* for second child, and of *cleaners* for house work; no support from extended family due to *geographical distance*; - One-off support from *government* to set up house to accommodate child’s disability (no other financial benefit as both parents in FT employment) | Regular | X | One-Off |
| 29 | Ian & Eva^**^ (MOD) | - Live in rented house - Regular income and travel support from the *government* due to *autistic* child - Previous daily support from *sister* who lived with family to look after autistic child; no other family support due to *geographical distance* | Regular | Regular | X |
| 30 | Vera & Tom^*^ (MOD) | - Live in own house - No family support network due to *geographical distance* | X | X | X |

Notes: *One of the parents born abroad, currently living and raising children in the UK: **Both parents born abroad, currently living and raising children in the UK; Severe Chronic Resource Scarcity: SVR; Moderate Chronic Resource Scarcity: MOD; Mild Chronic Resource Scarcity: MILD
